# Routine blood-based immune signatures in endogenous Cushing’s syndrome: associations with cortisol excess and selected complication burden

**DOI:** 10.3389/fendo.2026.1961986

**Published:** 2026-10-22

**Authors:** Yunjia Cai, Shuwen Tan, Siyuan Liu, Miao Wang, Tiemin Liu, Guixia Wang

**Affiliations:** 1 Department of Endocrinology and Metabolism, The First Hospital of Jilin University, Changchun, China; 2 State Key Laboratory of Genetic Engineering, School of Life Sciences, Fudan University, Shanghai, China; 3 Department of Obstetrics and Gynecology, Jilin Province People’s Hospital, Changchun, China

**Keywords:** Cushing’s syndrome, disease burden, eosinophil depletion, hypercortisolism, neutrophil-to-lymphocyte ratio, systemic immune-inflammation index

## Abstract

**Background:**

Routine blood-based markers capture glucocorticoid-related immune-cell redistribution, but whether these measures are associated with complementary biochemical measures of cortisol excess and clinical complication burden in endogenous Cushing’s syndrome remains unclear.

**Methods:**

We included 202 patients with endogenous Cushing’s syndrome. Seven routine blood-based immune markers were examined in relation to 24h urinary free cortisol (24h-UFC), midnight serum cortisol, and 8 a.m. serum cortisol. Selected immune markers were further evaluated in relation to clinical complications. An exploratory complication-domain burden score comprised four domains: electrolyte involvement, hypertension, diabetes, and fracture. An exploratory cortisol-related immune score (CRIS) combined neutrophil-to-lymphocyte ratio (NLR), eosinophil depletion, and low prognostic nutritional index (PNI).

**Results:**

All seven immune markers were significantly associated with each of the three biochemical cortisol measures after adjustment for age, sex, and aetiology, with NLR and absolute eosinophil count (AEC) showing the strongest and most consistent associations. For 24h-UFC, the adjusted coefficients were 0.647 for log-NLR and −0.687 for log(AEC + 0.01) per 1 SD increase. In primary complication analyses, significant associations after false-discovery rate (FDR) correction were observed for hypokalaemia, hypophosphataemia, diabetes, and fracture; for example, log-NLR was associated with hypophosphataemia (OR 2.86, 95% CI 1.39–6.33), while low PNI was associated with fracture (OR 2.52, 95% CI 1.60–3.96). Each 1 SD increase in CRIS was associated with a higher complication-domain burden score (IRR 1.20, 95% CI 1.07–1.35; *P* = 0.001).

**Conclusion:**

Routine blood-based immune markers were associated with complementary biochemical measures of cortisol excess and selected clinical complications in endogenous Cushing’s syndrome. Eosinophil depletion and increased NLR were consistently associated with all three cortisol measures, whereas associations with clinical complications varied by marker and outcome. CRIS provided an exploratory integrative summary of cortisol-related immune alterations and selected complication-domain burden but requires external validation.

## 1 Introduction

Endogenous Cushing’s syndrome is a rare but severe endocrine disorder resulting from prolonged exposure to excess cortisol (1–3). The effects of hypercortisolism extend across multiple organ systems, and patients may develop metabolic, electrolyte, skeletal, cardiovascular, infectious, and neuropsychiatric complications (4, 5). These complications account for much of the excess morbidity and mortality associated with the disease and may persist even after successful treatment of the underlying cause (1, 6). Assessment of disease burden therefore requires attention not only to cortisol concentrations but also to the clinical consequences of chronic cortisol excess (7, 8).

Glucocorticoid excess is associated with a characteristic pattern of circulating leukocytes, including increased neutrophil counts and reduced lymphocyte and eosinophil counts (9, 10). These changes are readily identified in routine blood tests. Several derived indices, including the neutrophil-to-lymphocyte ratio (NLR), systemic immune-inflammation index (SII), and systemic inflammation response index (SIRI), have been studied as indicators of systemic inflammation and disease severity in other clinical settings. The prognostic nutritional index (PNI), which incorporates serum albumin and lymphocyte count, has also been used to assess immune and nutritional status (11–15). Their clinical relevance in endogenous Cushing’s syndrome, however, remains uncertain.

Biochemical measures, including 24h urinary free cortisol (24h-UFC) and midnight serum cortisol, assess different aspects of cortisol excess. However, biochemical measures do not necessarily capture the full extent of the clinical consequences of chronic cortisol excess (8, 16, 17). Patients with similar cortisol measurements may differ substantially in the number and pattern of complications. Peripheral immune alterations may reflect both cortisol exposure and its systemic effects (8, 18). However, the associations of these markers with specific complications after accounting for biochemical cortisol measures remain less well characterised.

We examined routine blood-based immune markers in 202 patients with endogenous Cushing’s syndrome caused by adrenocortical adenoma (ACA), Cushing’s disease (CD), or ectopic adrenocorticotropic hormone (ACTH) syndrome. We assessed their associations with 24h-UFC, midnight serum cortisol, and 8AM serum cortisol, and examined whether individual markers were associated with prevalent electrolyte abnormalities, diabetes, and fracture after adjustment for 24h-UFC. We also examined associations with increased relative wall thickness (RWT) and evaluated an exploratory CRIS in relation to cortisol measures and selected complication-domain burden.

## 2 Materials and methods

### 2.1 Study design and population

This single-centre retrospective study included patients with endogenous Cushing’s syndrome admitted to the First Hospital of Jilin University between December 2012 and December 2022. Patients were hospitalised for diagnostic confirmation, aetiological evaluation, and treatment planning for suspected or established endogenous Cushing’s syndrome, with presenting manifestations including Cushingoid features, hypertension, weight gain, weakness or oedema, hyperglycaemia, menstrual disturbance, and pituitary-related symptoms such as headache or visual impairment. This cohort has been described previously in a study of clinical characteristics and postoperative recurrence (19); the present study addressed a distinct research question concerning blood count–derived immune markers and disease burden.

Endogenous Cushing’s syndrome was diagnosed according to the Endocrine Society clinical practice guideline (16). Biochemical hypercortisolism was confirmed by elevated 24h-UFC on at least two collections and failure of serum cortisol to suppress below 50 nmol/L after an overnight 1-mg dexamethasone suppression test. Individual post-dexamethasone cortisol concentrations were not systematically available in the analytical dataset and were therefore not included as study endpoints. For the present analyses, 24h-UFC referred to the measurement available at the index admission; therefore, analysable 24h-UFC data were available in 173 patients despite prior biochemical testing used for diagnostic confirmation. After confirmation of endogenous hypercortisolism, aetiology was determined using plasma ACTH concentrations, adrenal or pituitary imaging, and additional dynamic testing as clinically indicated. Patients were subsequently classified as having ACA, CD, or ectopic ACTH syndrome (EAS). Patients were excluded if they had received long-term exogenous glucocorticoid treatment, had long-term heavy alcohol consumption (≥ 30 g/day for men or ≥ 15 g/day for women), had pseudo-Cushing’s syndrome associated with depression or obesity, severe organ dysfunction, active inflammatory or autoimmune disease, haematological disorders, malignancy, clinically significant malnutrition, or were receiving medications known to substantially affect peripheral blood cell counts. For patients with multiple admissions, only data from the first admission before definitive treatment were included. The final cohort comprised 202 patients, including 71 with ACA, 122 with CD, and 9 with EAS.

The study protocol was reviewed and approved by the Ethics Committee of the First Hospital of Jilin University (approval no. 2024-566) and was conducted in accordance with the Declaration of Helsinki. The requirement for informed consent was waived by the same ethics committee because of the retrospective study design.

### 2.2 Data collection and clinical definitions

Demographic, clinical, laboratory, and imaging data were obtained from electronic medical records at the index admission before definitive treatment. Variables included age, sex, body mass index, disease duration, blood pressure, documented hypertension status, aetiology, midnight serum cortisol, 8AM serum cortisol, 24h-UFC, ACTH, albumin, potassium, phosphate, blood count parameters, and Cushing’s syndrome–related complications. Complete blood counts were measured in the central clinical laboratory using the same automated haematology analyser throughout the study period. The laboratory reference intervals, reporting units, and analytical procedures remained unchanged from 2012 to 2022. AECs were reported in units of × 10^9^/L at a resolution of 0.01 × 10^9^/L. Midnight and 8AM serum cortisol, as well as 24h-UFC, were measured using chemiluminescent immunoassays in the hospital clinical laboratory. The reference interval for 8 a.m. serum cortisol was 240–619 nmol/L, and that for 24h-UFC was 108–961 nmol/24 h. The assay methodology remained unchanged during the study period. For clinical interpretability, 24h-UFC was also expressed as fold upper limit of normal (×ULN), calculated by dividing the measured 24h-UFC value by the upper reference limit of 961 nmol/24h.

The individual complication outcomes examined in marker-specific analyses were hypokalaemia, hypophosphataemia, diabetes, fracture, and increased RWT. These outcomes were ascertained from clinical history, laboratory results, and imaging available at the index admission before definitive treatment and were analysed as prevalent complications rather than incident events. Hypokalaemia and hypophosphataemia were defined as serum potassium <3.5 mmol/L and phosphate <0.8 mmol/L, respectively. Diabetes was defined by a documented diagnosis, glucose-lowering treatment, fasting plasma glucose ≥7.0 mmol/L, 2h plasma glucose ≥11.1 mmol/L, or symptomatic random plasma glucose ≥11.1 mmol/L. Diabetes was counted as one affected domain whenever these criteria were met at the index admission, irrespective of whether diabetes had been diagnosed before recognition of Cushing’s syndrome, because the score was intended to summarise concurrent complication burden rather than attribute individual complications causally to hypercortisolism. Fracture status was abstracted from documented clinical history and available imaging reports. Dedicated systematic screening for asymptomatic vertebral fractures was not part of the retrospective outcome ascertainment. RWT, an echocardiographic measure of left ventricular geometry, was obtained from transthoracic echocardiographic examinations available at the index admission before definitive treatment. RWT data were available for 171 of 202 patients (84.7%), and increased RWT was defined as RWT ≥0.45.

The selected complication-domain burden score was constructed as an exploratory summary of four systematically ascertainable domains: electrolyte involvement, hypertension, diabetes, and fracture. Hypokalaemia and hypophosphataemia were combined into a single electrolyte domain, and hypertension was defined by a documented diagnosis in the medical record. One point was assigned to each affected domain, yielding a score ranging from 0 to 4 that represented the number of affected domains and was analysed as a count outcome. For exploratory binary analyses, multidomain complication involvement was defined as involvement of at least two domains, indicating involvement of more than one clinical domain rather than a validated severity threshold. The score was not intended to replace a validated measure of overall Cushing’s syndrome severity. Previous cardiovascular events were not included because of their low frequency, thromboembolic events were not systematically captured, and RWT was analysed separately because echocardiographic data were incomplete.

Documented infection was identified from diagnoses, microbiological findings, or anti-infective treatment during the index admission. CRP and procalcitonin were available in only five and two patients, respectively, and were therefore not included in the analyses.

### 2.3 Blood count–derived immune markers and CRIS construction

Routine blood count–derived indices included NLR, the platelet-to-lymphocyte ratio (PLR), the lymphocyte-to-monocyte ratio (LMR), SII, SIRI, and PNI. These were calculated as follows: NLR = neutrophils/lymphocytes, PLR = platelets/lymphocytes, LMR = lymphocytes/monocytes, SII = platelets × neutrophils/lymphocytes, SIRI = neutrophils × monocytes/lymphocytes, and PNI = albumin (g/L) + 5 × lymphocyte count (10^9^/L). The definitions of SII and SIRI followed their original descriptions (13, 15), and PNI was calculated according to the formula proposed by Onodera et al. (20). The original studies were conducted in hepatocellular carcinoma (SII; cut-off 330), advanced pancreatic cancer receiving chemotherapy (SIRI; cut-off 1.8), and gastrointestinal surgical patients (PNI; thresholds of 45 and 40). Because these thresholds were setting-specific and have not been validated in Cushing’s syndrome, the indices were analysed as continuous variables in the present study. Absolute neutrophil count (ANC), absolute lymphocyte count (ALC), and AEC were obtained directly from routine blood tests. PLR and LMR were included only for descriptive comparisons across aetiological groups and were not carried forward to the cortisol or complication association analyses.

24h-UFC was used to characterise integrated urinary cortisol excretion over 24h and as the biochemical covariate in the primary individual-marker complication models. Midnight serum cortisol was included in the main cortisol association analyses to assess nocturnal cortisol elevation, a feature of disrupted circadian secretion. Serum cortisol measured at 8 a.m. was included as an additional routinely available measurement of circulating cortisol at the index evaluation; it was not interpreted as a validated measure of overall cortisol burden. All three measures were analysed as continuous variables. For exploratory categorical analyses, upper-quartile 8 a.m. serum cortisol and upper-quartile 24h-UFC were defined as values ≥ 747.30 nmol/L and ≥ 4.11 ×ULN, respectively. These cohort-specific thresholds were used for internal stratification rather than as established clinical cut-offs.

The exploratory CRIS was constructed from log-transformed NLR, eosinophil depletion, and low PNI. Eosinophil depletion was expressed as −log(AEC + 0.01), and low PNI as −PNI. Each component was standardised, and CRIS was calculated as: CRIS = [Z(log NLR) + Z(−log(AEC + 0.01)) + Z(−PNI)]/3. Higher values indicate a more pronounced cortisol-related peripheral immune profile. CRIS was defined *de novo* as an equally weighted mean of three standardised components representing neutrophil–lymphocyte imbalance, eosinophil depletion, and reduced PNI, and was considered exploratory rather than a validated diagnostic or clinical decision score. Division by three retained the score on the scale of the average component without altering the relative weights.

### 2.4 Statistical analysis

Analyses were based on available data for each outcome, with variable availability summarised in **Supplementary Table 1**. Characteristics of patients with and without available 24h-UFC measurements were compared, and missing 24h-UFC values were further addressed using multiple imputation by chained equations with 50 imputed datasets. Estimates were combined using Rubin’s rules. Continuous variables are presented as mean ± standard deviation (SD) or median (interquartile range), and categorical variables as number and percentage. Aetiological groups were compared using analysis of variance, the Kruskal–Wallis test, χ² test, or Fisher’s exact test, with Dunn’s test for *post hoc* comparisons.

Associations between immune markers and each continuous cortisol measure (24h-UFC, midnight serum cortisol, and 8 a.m. serum cortisol) were examined using Spearman correlation and multivariable linear regression adjusted for age, sex, and aetiology. For linear regression, all three cortisol outcomes were natural log-transformed. All seven markers (ANC, ALC, AEC, NLR, SII, SIRI, and PNI) were evaluated in the regression analyses. ANC, ALC, and PNI were modelled on their original scales; AEC was modelled as log(AEC + 0.01), whereas NLR, SII, and SIRI were natural log-transformed. All immune predictors were standardised before regression. AEC, NLR, SII, SIRI, and PNI were subsequently carried forward as the five principal risk-oriented markers for the downstream complication analyses.

Exploratory receiver operating characteristic (ROC) analyses compared clinical, immune, and combined models in common complete-case samples. Areas under the ROC curve (AUCs) were compared using paired DeLong tests, and internal validation was performed with 1,000 bootstrap resamples to estimate optimism-corrected AUCs, calibration slopes, and Brier scores. Individual-marker complication analyses excluded patients with EAS and were adjusted for age, sex, aetiology, and log-transformed 24h-UFC (×ULN). Patients with EAS were excluded from the primary individual-marker complication analyses because of the very small subgroup size and the extreme cortisol and complication profiles in this group, which could disproportionately influence multivariable estimates. Conventional logistic regression was used for hypokalaemia, diabetes, and fracture, whereas Firth penalised logistic regression was used for hypophosphataemia and increased RWT. Models without 24h-UFC adjustment and analyses excluding documented infection were examined as sensitivity analyses, with Benjamini–Hochberg FDR correction applied across the prespecified marker–outcome comparisons. Associations of CRIS with the selected complication-domain burden score were examined using Poisson regression, with adjustment for age, sex, and aetiology. Multidomain complication involvement, defined as involvement of at least two domains, was examined as an exploratory binary outcome using Firth penalised logistic regression because of complete separation in the EAS subgroup. A sensitivity model additionally adjusted for log-transformed 24h-UFC (×ULN). CRIS was compared with log-NLR using DeLong testing, Akaike information criterion, likelihood-ratio testing, and tenfold cross-validation; inter-component correlations and variance inflation factors were also examined. Additional sensitivity analyses assessed alternative AEC offsets, exclusion of EAS or documented infection, and aetiology-specific models. Exploratory mediation analyses evaluated the relationships among 24h-UFC, immune markers, and electrolyte complications using 1,000 bootstrap resamples. Analyses were performed using R 4.5.3 and GraphPad Prism 9.5. All tests were two-sided, with *P* < 0.05 considered significant.

## 3 Results

### 3.1 Baseline characteristics and aetiology-specific immune profiles

A total of 202 patients were included: 71 with ACA, 122 with CD, and 9 with EAS; the median age was 46 years and 176 (87.1%) were women. Patients with EAS had higher 24h-UFC, midnight and 8 a.m. serum cortisol, and ACTH concentrations, lower albumin, more documented infections, and the highest median complication-domain burden score (**Table 1**). The study flow and analytical framework are shown in **Figure 1**.

**Table 1:** Study-relevant baseline characteristics of patients with endogenous Cushing’s syndrome according to aetiology.

Variable	Overall	ACA	CD	EAS	*P*-value
No. of patients	202	71	122	9	—
Age, years	46 (32, 54)	42 (30.5, 50.5)	47 (34, 56)	56 (49, 67)	< 0.001
Female sex, n/N (%)	176/202 (87.1)	68/71 (95.8)	103/122 (84.4)	5/9 (55.6)	0.001
BMI, kg/m²	25.70 (23.03, 27.84)	25.78 (22.88, 27.80)	26.05 (23.44, 29.06)	23.66 (23.38, 25.26)	0.345
Disease duration, months	12 (12, 36)	12 (12, 24)	18 (12, 36)	3.5 (1, 8.25)	0.003
24h-UFC, ×ULN	2.09 (1.18, 4.11)	1.98 (1.49, 3.23)	2.11 (1.03, 4.22)	20.63 (16.18, 34.03)	0.001
Midnight serum cortisol, nmol/L	460.14 (288.19, 609.08)	512.78 (390.10, 594.83)	380.00 (203.80, 600.37)	1693.93 (1390.85, 1754.63)	< 0.001
8AM serum cortisol, nmol/L	543.37 (403.76, 747.30)	563.34 (462.78, 656.73)	526.13 (374.71, 783.99)	1671.86 (1655.31, 1754.63)	< 0.001
ACTH, pg/ml	6.86 (0.22, 23.05)	0.22 (0.22, 0.33)	21.52 (13.85, 26.49)	60.05 (37.84, 80.84)	< 0.001
Albumin, g/L	38.70 (35.75, 41.30)	38.00 (35.30, 41.70)	39.20 (36.50, 41.30)	31.40 (28.30, 33.10)	0.001
Electrolyte-domain involvement, n/N (%)	83/185 (44.9)	26/62 (41.9)	48/114 (42.1)	9/9 (100.0)	0.002
Hypertension, n/N (%)	145/202 (71.8)	55/71 (77.5)	82/122 (67.2)	8/9 (88.9)	0.203
Diabetes, n/N (%)	71/202 (35.1)	16/71 (22.5)	49/122 (40.2)	6/9 (66.7)	0.005
Fracture, n/N (%)	57/202 (28.2)	15/71 (21.1)	40/122 (32.8)	2/9 (22.2)	0.236
Selected complication-domain burden score, median (IQR)	2 (1, 3)	1 (1, 2)	2 (1, 3)	3 (2, 3)	0.009
Multidomain complication involvement, n/N (%)	103/185 (55.7)	29/62 (46.8)	65/114 (57.0)	9/9 (100.0)	0.005
Increased RWT, n/N (%)	25/171 (14.6)	13/65 (20.0)	12/98 (12.2)	0/8 (0.0)	0.233
Documented infection, n/N (%)	14/202 (6.9)	3/71 (4.2)	7/122 (5.7)	4/9 (44.4)	0.002

Continuous variables are presented as median and interquartile range, and categorical variables as number/number with available data (percentage). The selected complication-domain burden score included four domains: electrolyte-domain involvement, hypertension, diabetes, and fracture. Electrolyte-domain involvement was defined as the presence of hypokalaemia and/or hypophosphataemia. One point was assigned for each affected domain, yielding a score ranging from 0 to 4. Multidomain complication involvement was defined as involvement of at least two domains. Group comparisons were performed using the Kruskal–Wallis test for continuous or ordinal variables and the χ² test or Fisher’s exact test for categorical variables, as appropriate. ACA, adrenocortical adenoma; CD, Cushing’s disease; EAS, ectopic ACTH syndrome; BMI, body mass index; UFC, urinary free cortisol; ACTH, adrenocorticotropic hormone; RWT, relative wall thickness; IQR, interquartile range; ULN, upper limit of normal.

**Figure 1:**
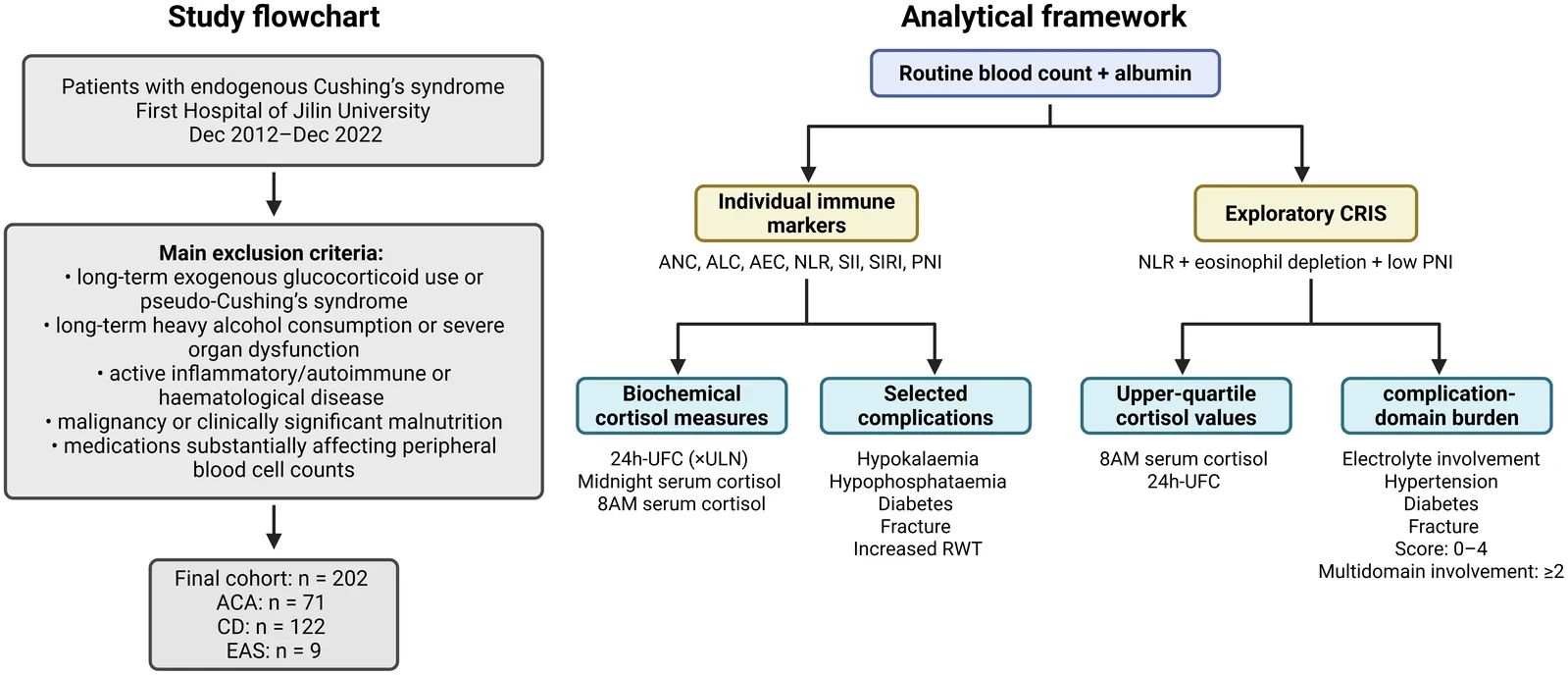
Study design and analytical framework. Flowchart showing cohort selection and the analytical framework for immune-marker and exploratory CRIS analyses in 202 patients with endogenous Cushing’s syndrome. The complication-domain burden score comprised electrolyte involvement, hypertension, diabetes, and fracture (range: 0–4); RWT was analysed separately. ACA, adrenocortical adenoma; CD, Cushing’s disease; EAS, ectopic ACTH syndrome; CRIS, cortisol-related immune score; RWT, relative wall thickness; UFC, urinary free cortisol.

Blood count-derived immune profiles differed by aetiology (**Table 2**; **Figure 2**). Total white blood cell count (WBC) was similar across groups, whereas patients with EAS generally showed higher ANC and NLR and lower ALC, AEC, and PNI. SIRI differed across groups, while the borderline difference in SII did not remain significant after FDR correction.

**Table 2:** Blood count-derived immune indices across aetiological subgroups of endogenous Cushing’s syndrome.

Marker	ACA	CD	EAS	*P*-value	FDR *q*-value
WBC, ×10^9^/L	8.28 (7.36, 9.34)	7.77 (6.20, 9.77)	9.80 (7.08, 10.63)	0.115	0.115
ANC, ×10^9^/L	6.22 (4.80, 7.16)	5.48 (3.95, 7.24)	8.57 (5.24, 9.17)	0.027	0.034
ALC, ×10^9^/L	1.58 (1.21, 1.98)	1.73 (1.25, 2.20)	1.04 (0.69, 1.38)	0.003	0.006
AEC, ×10^9^/L	0.03 (0.02, 0.07)	0.04 (0.01, 0.09)	0.00 (0.00, 0.00)	< 0.001	0.001
Platelet count, ×10^9^/L	241 (210, 296)	232 (201, 260)	136 (101, 178)	< 0.001	0.002
NLR	3.73 (2.95, 5.11)	3.32 (2.03, 5.02)	6.08 (4.49, 14.30)	0.001	0.003
PLR	155.70 (132.07, 196.21)	130.65 (105.11, 181.23)	118.84 (94.66, 219.57)	0.020	0.032
LMR	2.79 (2.13, 3.80)	3.39 (2.40, 5.07)	3.29 (2.66, 3.47)	0.028	0.034
SII	944.86 (657.16, 1242.57)	761.01 (480.03, 1161.09)	1020.38 (494.11, 1945.39)	0.050	0.055
SIRI	2.21 (1.33, 3.14)	1.55 (1.01, 2.83)	2.19 (1.55, 3.38)	0.016	0.030
PNI	46.00 (42.10, 50.25)	47.75 (43.75, 51.95)	36.60 (31.80, 39.65)	< 0.001	< 0.001

Data are presented as median (interquartile range). Group comparisons were performed using the Kruskal–Wallis test, and FDR q values were calculated using the Benjamini–Hochberg method. ACA, adrenocortical adenoma; CD, Cushing’s disease; EAS, ectopic adrenocorticotropic hormone syndrome; WBC, white blood cell count; ANC, absolute neutrophil count; ALC, absolute lymphocyte count; AEC, absolute eosinophil count; NLR, neutrophil-to-lymphocyte ratio; PLR, platelet-to-lymphocyte ratio; LMR, lymphocyte-to-monocyte ratio; SII, systemic immune-inflammation index; SIRI, systemic inflammation response index; PNI, prognostic nutritional index; FDR, false discovery rate.

**Figure 2:**
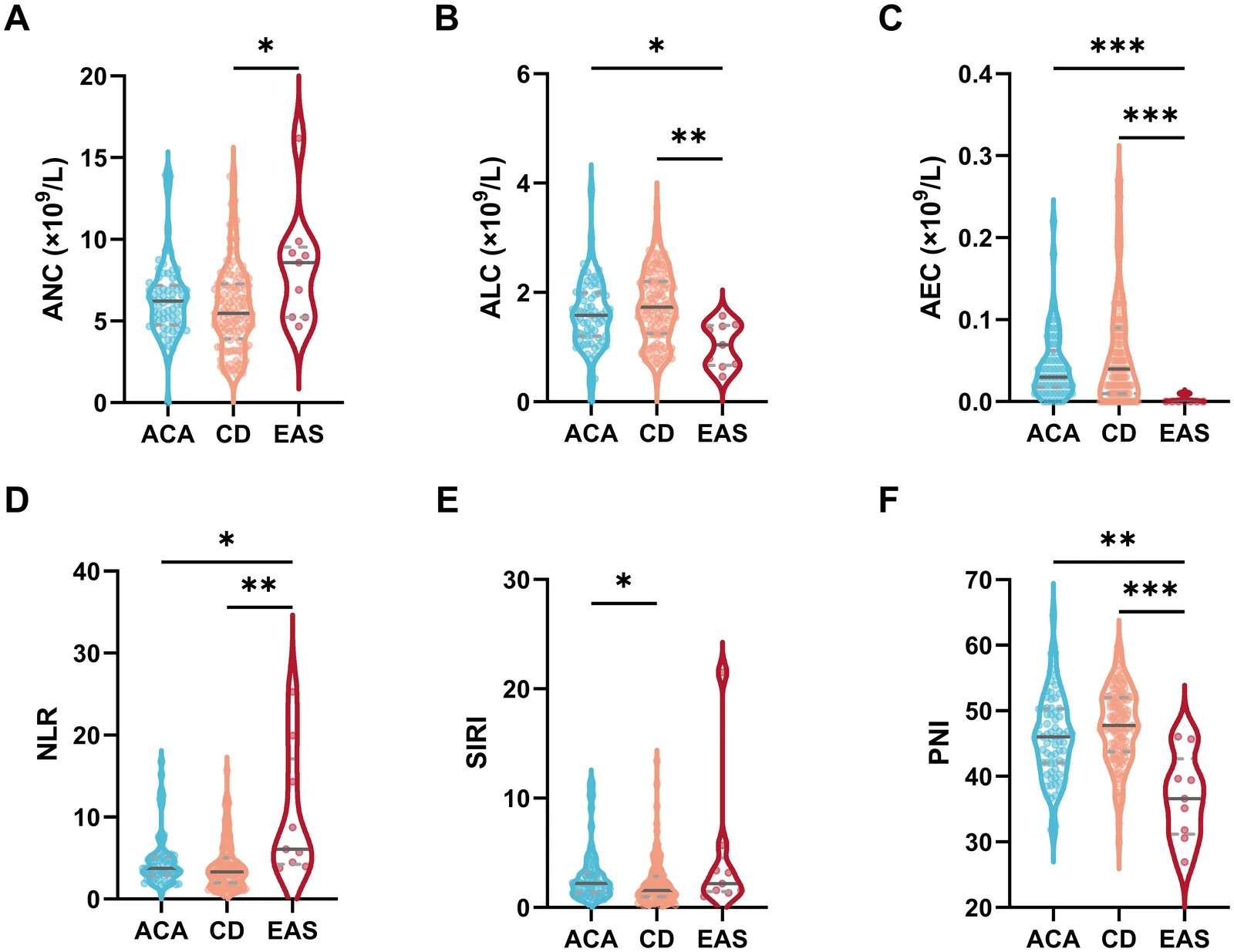
Blood count–derived immune profiles across aetiological subgroups of endogenous Cushing’s syndrome. The figure shows the distributions of **(A)** absolute neutrophil count (ANC), **(B)** absolute lymphocyte count (ALC), **(C)** absolute eosinophil count (AEC), **(D)** neutrophil-to-lymphocyte ratio (NLR), **(E)** systemic inflammation response index (SIRI), and **(F)** prognostic nutritional index (PNI) across aetiological subgroups. Each point represents one patient. Violin plots show the data distribution, and horizontal lines indicate the median and interquartile range. Group comparisons were performed using the Kruskal–Wallis test, and pairwise comparisons were performed using Dunn’s multiple comparisons test. ACA, adrenocortical adenoma; CD, Cushing’s disease; EAS, ectopic ACTH syndrome. *P < 0.05, **P < 0.01, ***P < 0.001.

24h-UFC measurements were available in 173 of 202 patients (85.6%). Patients without available 24h-UFC were more frequently classified as having ACA and had a lower prevalence of fracture, whereas most other clinical and immune characteristics were similar between groups (**Supplementary Table S2**).

### 3.2 Blood count-derived immune markers are associated with biochemical cortisol measures

Blood count-derived immune markers showed consistent associations across all three biochemical cortisol measures (**Figure 3**; **Table 3**). ANC, NLR, SII, and SIRI were positively correlated with 24h-UFC, midnight serum cortisol, and 8 a.m. serum cortisol, whereas ALC, AEC, and PNI were inversely correlated with all three measures; all correlations remained significant after FDR correction. NLR and AEC showed the strongest and most consistent associations. NLR correlated positively with 24h-UFC (ρ = 0.639), midnight serum cortisol (ρ = 0.640), and 8AM serum cortisol (ρ = 0.627), whereas AEC correlated inversely with the corresponding measures (ρ = −0.715, −0.608, and −0.619, respectively). The largest absolute unadjusted correlation was observed between AEC and 24h-UFC.

**Figure 3:**
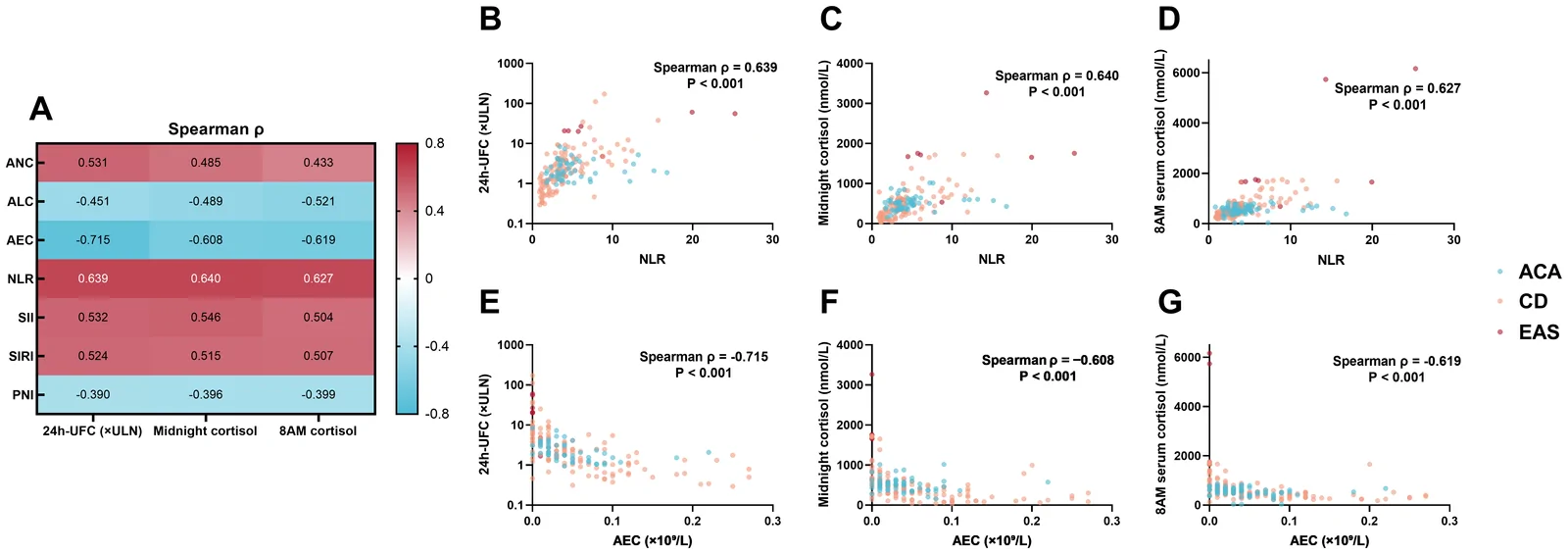
Blood count–derived immune markers are associated with complementary biochemical cortisol measures. **(A)** Correlation heatmap showing Spearman correlation coefficients between blood count-derived immune markers and 24h-UFC, midnight serum cortisol, and 8 a.m. serum cortisol. Red indicates positive correlations and blue indicates negative correlations. **(B–D)** Correlations of NLR with 24h-UFC, midnight serum cortisol, and 8 a.m. serum cortisol, respectively. **(E–G)** Correlations of AEC with 24h-UFC, midnight serum cortisol, and 8 a.m. serum cortisol, respectively. Each point represents one patient and is coloured according to aetiology. Correlations were assessed using Spearman correlation analysis. For visualisation, 24h-UFC is shown on a log_10_ scale. All correlations shown in the heatmap remained significant after false-discovery rate correction. ACA, adrenocortical adenoma; CD, Cushing’s disease; EAS, ectopic ACTH syndrome; ANC, absolute neutrophil count; ALC, absolute lymphocyte count; AEC, absolute eosinophil count; NLR, neutrophil-to-lymphocyte ratio; SII, systemic immune-inflammation index; SIRI, systemic inflammation response index; PNI, prognostic nutritional index; UFC, urinary free cortisol.

**Table 3:** Associations between blood count-derived immune markers and biochemical cortisol measures.

(A) 24h-UFC ×ULN)					
Marker	*n*	Spearman ρ	FDR *q*-value	Adjusted β (95% CI)	Adjusted *P*-value
ANC	173	0.531	< 0.001	0.489 (0.352 to 0.626)	< 0.001
ALC	173	−0.451	< 0.001	−0.434 (−0.578 to −0.290)	< 0.001
AEC	173	−0.715	< 0.001	−0.687 (−0.808 to −0.565)	< 0.001
NLR	173	0.639	< 0.001	0.647 (0.520 to 0.773)	< 0.001
SII	173	0.532	< 0.001	0.562 (0.429 to 0.696)	< 0.001
SIRI	173	0.524	< 0.001	0.537 (0.402 to 0.672)	< 0.001
PNI	171	−0.390	< 0.001	−0.448 (−0.599 to −0.297)	< 0.001
(B) Midnight serum cortisol					
Marker	*n*	Spearman ρ	FDR *q*-value	Adjusted β (95% CI)	Adjusted *P*-value
ANC	177	0.485	< 0.001	0.352 (0.253 to 0.450)	< 0.001
ALC	177	−0.489	< 0.001	−0.273 (−0.377 to −0.169)	< 0.001
AEC	177	−0.608	< 0.001	−0.409 (−0.506 to −0.313)	< 0.001
NLR	177	0.640	< 0.001	0.464 (0.372 to 0.556)	< 0.001
SII	177	0.546	< 0.001	0.421 (0.327 to 0.515)	< 0.001
SIRI	177	0.515	< 0.001	0.389 (0.294 to 0.485)	< 0.001
PNI	176	−0.396	< 0.001	−0.260 (−0.373 to −0.148)	< 0.001
(C) 8AM serum cortisol					
Marker	*n*	Spearman ρ	FDR *q*-value	Adjusted β (95% CI)	Adjusted *P*-value
ANC	193	0.433	< 0.001	0.218 (0.136 to 0.300)	< 0.001
ALC	193	−0.521	< 0.001	−0.233 (−0.315 to −0.151)	< 0.001
AEC	193	−0.619	< 0.001	−0.280 (−0.361 to −0.198)	< 0.001
NLR	193	0.627	< 0.001	0.315 (0.236 to 0.393)	< 0.001
SII	193	0.504	< 0.001	0.280 (0.201 to 0.359)	< 0.001
SIRI	193	0.507	< 0.001	0.270 (0.191 to 0.350)	< 0.001
PNI	191	−0.399	< 0.001	−0.227 (−0.317 to −0.138)	< 0.001

Spearman correlations were calculated using the original marker values, with false-discovery rate (FDR) *q*-values calculated using the Benjamini–Hochberg method. Multivariable linear regression models were adjusted for age, sex, and aetiology. All three cortisol measures were natural log-transformed for regression analyses. ANC, ALC, and PNI were modelled on their original scales; AEC was modelled as log(AEC + 0.01), whereas NLR, SII, and SIRI were natural log-transformed. All immune predictors were standardised before regression, and adjusted β values represent changes in the log-transformed cortisol measure per 1 SD increase in each marker. ANC, absolute neutrophil count; ALC, absolute lymphocyte count; AEC, absolute eosinophil count; NLR, neutrophil-to-lymphocyte ratio; SII, systemic immune-inflammation index; SIRI, systemic inflammation response index; PNI, prognostic nutritional index; UFC, urinary free cortisol; FDR, false-discovery rate; CI, confidence interval; ULN, upper limit of normal.

After adjustment for age, sex, and aetiology, all seven immune markers remained significantly associated with each of the three log-transformed cortisol measures (**Table 3**). For 24h-UFC, log(AEC + 0.01) and log-NLR showed the largest absolute adjusted coefficients (β = −0.687 and 0.647 per 1 SD increase, respectively), while log-NLR showed the largest adjusted coefficient for midnight serum cortisol (β = 0.464).

AEC was recorded as 0.00 ×10^9^/L in 35 of 202 patients (17.3%), but its associations with 8AM serum cortisol and 24h-UFC were robust to alternative offsets and categorical parameterisation (**Supplementary Table S3**). Associations between immune markers and these cortisol measures were also directionally consistent across calendar periods, with no marker-by-period interaction remaining significant after FDR correction (**Supplementary Table S4**). Exploratory ROC analyses are presented in **Supplementary Figure S1**; **Supplementary Tables S5**, **S6**.

### 3.3 Individual immune markers are associated with selected complications

Primary complication analyses excluded patients with EAS and were adjusted for age, sex, aetiology, and log-transformed 24h-UFC. After FDR correction, significant associations remained for hypokalaemia, hypophosphataemia, diabetes, and fracture, whereas none of the associations with increased RWT remained significant (**Figure 4**; **Supplementary Table S7**).

**Figure 4:**
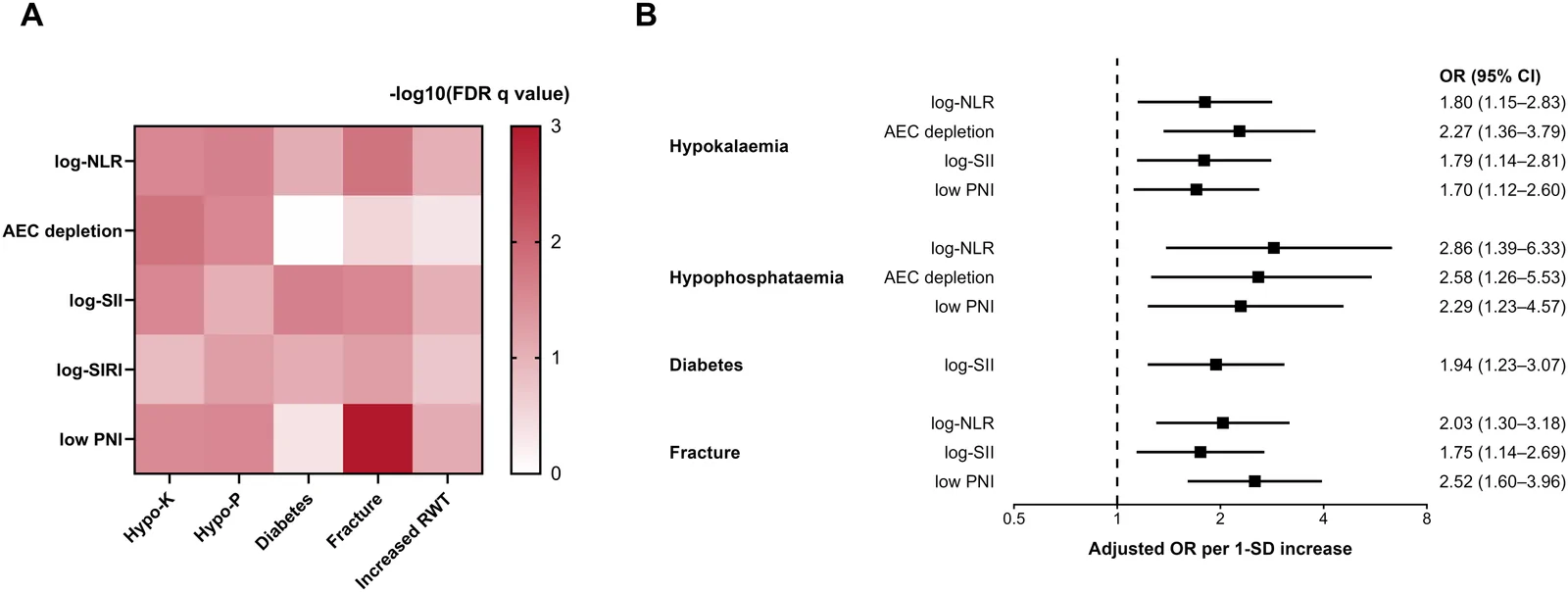
Associations of blood count–derived immune markers with selected complications. **(A)** Heatmap showing the statistical significance of associations between risk-oriented immune markers and selected complications, expressed as −log_10_(FDR *q*-value). Benjamini–Hochberg false-discovery rate correction was applied across all 25 prespecified marker–outcome comparisons. **(B)** Forest plot showing adjusted odds ratios (ORs) and 95% confidence intervals (CIs) for associations that remained significant after FDR correction. Primary analyses excluded patients with ectopic ACTH syndrome (EAS) and were adjusted for age, sex, aetiology, and log-transformed 24h urinary free cortisol (24h-UFC, ×ULN). ORs are reported per 1 SD increase in each risk-oriented immune marker. In the primary analyses underlying **(A)** conventional logistic regression was used for hypokalaemia, diabetes, and fracture, whereas Firth penalised logistic regression was used for hypophosphataemia and increased relative wall thickness (RWT) because of the limited number of events. NLR, neutrophil-to-lymphocyte ratio; AEC, absolute eosinophil count; SII, systemic immune-inflammation index; SIRI, systemic inflammation response index; PNI, prognostic nutritional index; FDR, false-discovery rate; ULN, upper limit of normal. Hypo-K, hypokalaemia; Hypo-P, hypophosphataemia.

For hypokalaemia, log-NLR, eosinophil depletion, log-SII, and low PNI were associated with higher odds, with adjusted ORs ranging from 1.70 to 2.27. Hypophosphataemia was associated with log-NLR (OR 2.86, 95% CI 1.39–6.33), eosinophil depletion (OR 2.58, 95% CI 1.26–5.53), and low PNI (OR 2.29, 95% CI 1.23–4.57). Diabetes was associated with log-SII (OR 1.94, 95% CI 1.23–3.07), whereas fracture was associated with log-NLR, log-SII, and low PNI, with the strongest association observed for low PNI (OR 2.52, 95% CI 1.60–3.96).

Sensitivity analyses without 24h-UFC adjustment and after excluding patients with documented infection showed broadly similar patterns, although log-SII was additionally associated with increased RWT after FDR correction in the latter analysis (**Supplementary Table S7**). Multiple-imputation analyses addressing missing 24h-UFC values also yielded similar results (**Supplementary Table S8**). Exploratory mediation analyses identified significant indirect effects for several markers, most consistently for eosinophil depletion and log-NLR in the associations of 24h-UFC with hypokalaemia and hypophosphataemia (**Supplementary Table S9**).

### 3.4 Exploratory CRIS is associated with cortisol measures and complication-domain burden

The primary CRIS analysis included 182 patients. Higher CRIS quartiles were associated with higher frequencies of upper-quartile 24h-UFC and upper-quartile 8 a.m. serum cortisol, as well as greater selected complication-domain burden (**Table 4**; **Figure 5**). From Q1 to Q4, the mean burden score increased from 1.50 ± 1.03 to 2.47 ± 1.14, multidomain complication involvement from 39.1% to 77.8%, upper-quartile 8AM serum cortisol from 2.3% to 69.8%, and upper-quartile 24h-UFC from 5.0% to 61.5% (all *P* for trend < 0.001). After adjustment for age, sex, and aetiology, each 1 SD increase in CRIS was associated with a higher complication-domain burden score (IRR 1.20, 95% CI 1.07–1.35; *P* = 0.001) and with multidomain complication involvement (OR 1.90, 95% CI 1.32–2.82; *P* < 0.001).

**Table 4:** CRIS quartiles in relation to upper-quartile cortisol values and selected complication-domain burden.

CRIS quartile	*n*	Selected complication-domain burden score	Multidomain complication involvement, *n*/*N* (%)	Upper-quartile 8 a.m. serum cortisol, *n*/*N* (%)	Upper-quartile 24h-UFC, *n/N* (%)
Q1	46	1.50 ± 1.03	18/46 (39.1)	1/43 (2.3)	2/40 (5.0)
Q2	46	1.37 ± 0.90	19/46 (41.3)	5/45 (11.1)	4/37 (10.8)
Q3	45	2.00 ± 1.07	30/45 (66.7)	10/43 (23.3)	10/39 (25.6)
Q4	45	2.47 ± 1.14	35/45 (77.8)	30/43 (69.8)	24/39 (61.5)
P for trend	—	< 0.001	< 0.001	< 0.001	< 0.001
Association per 1 SD increase in CRIS					
Outcome		*n*	Effect estimate	95% CI	*P*-value
Selected complication-domain burden score		182	IRR 1.20	1.07–1.35	0.001
Multidomain complication involvement		182	OR 1.90	1.32–2.82	< 0.001

CRIS quartiles were defined in patients with available CRIS and complication-domain burden data. The burden score comprised four domains: electrolyte involvement, hypertension, diabetes, and fracture, with one point assigned to each domain range: 0–4. Multidomain complication involvement was defined as involvement of at least two domains and was treated as an exploratory binary outcome. Continuous variables are presented as mean ± standard deviation and categorical variables as *n*/*N* %. *P*-values for trend were obtained by modelling CRIS quartiles as an ordinal variable. Associations per 1 SD increase in CRIS were adjusted for age, sex, and aetiology; Poisson regression was used for the burden score and Firth penalised logistic regression for multidomain complication involvement. CRIS, cortisol-related immune score; UFC, urinary free cortisol; OR, odds ratio; IRR, incidence rate ratio; CI, confidence interval.

**Figure 5:**
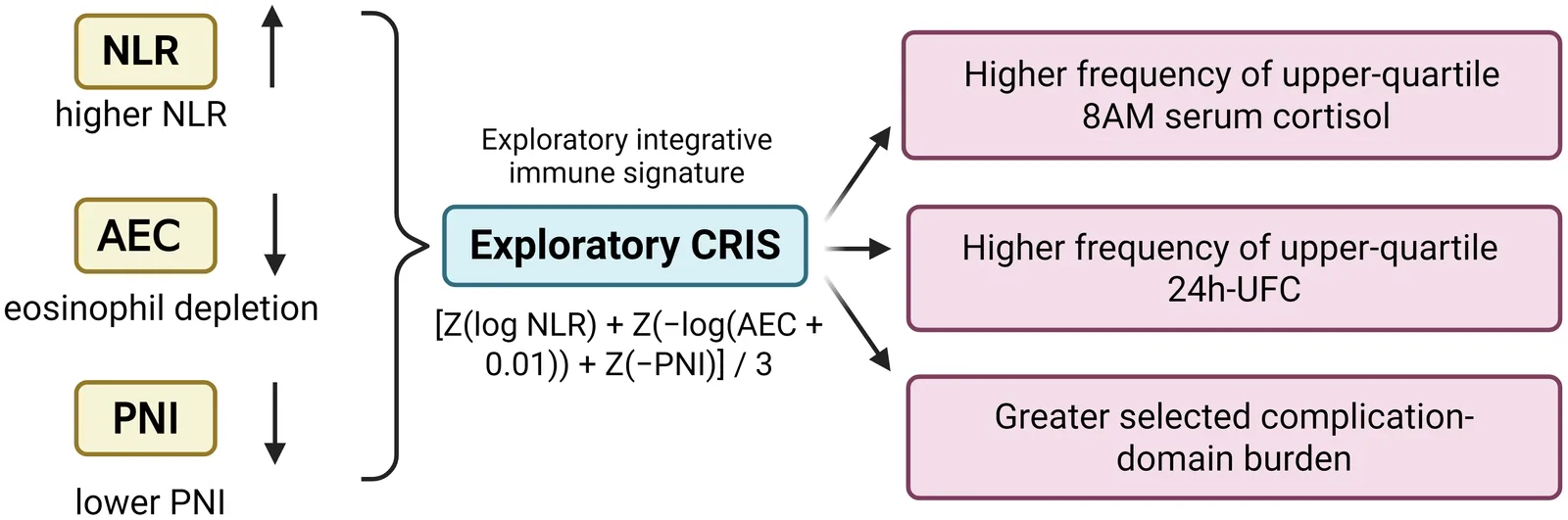
Construction of the exploratory CRIS and its associations with cortisol measures and complication-domain burden. CRIS was constructed as an equal-weight composite of three risk-oriented immune features: higher neutrophil-to-lymphocyte ratio (NLR), eosinophil depletion, and lower prognostic nutritional index (PNI), calculated as [Z(log NLR) + Z(−log(AEC + 0.01)) + Z(−PNI)]/3. Higher CRIS values were associated with higher frequencies of upper-quartile 8 a.m. serum cortisol and upper-quartile 24h urinary free cortisol (24h-UFC), as well as greater selected complication-domain burden. CRIS should be interpreted as an exploratory integrative immune signature rather than a validated clinical risk score. AEC, absolute eosinophil count; CRIS, cortisol-related immune score.

The association with the complication-domain burden score remained significant after additional adjustment for 24h-UFC (*n* = 155; IRR 1.29, 95% CI 1.09–1.51; *P* = 0.003), after excluding EAS or documented infection, and in the CD-only subgroup, but not in the ACA subgroup (**Supplementary Table S10**). Results were also robust to alternative standard-error estimation and AEC offsets, with a similar overall pattern for multidomain complication involvement (**Supplementary Table S11**).

CRIS and each of its individual components were associated with multidomain complication involvement (**Supplementary Table S12**). In the subset comprising patients with ACA or CD, CRIS showed no discrimination advantage over log-NLR (AUC 0.737 vs. 0.738; DeLong *P* = 0.943), and adding CRIS to log-NLR did not significantly improve model fit (likelihood-ratio *P* = 0.290); tenfold cross-validated AUCs were also similar (0.711 vs. 0.706; **Supplementary Table S13**).

## 4 Discussion

Cushing’s syndrome is a systemic disorder associated with substantial morbidity and excess mortality rather than simply a state of biochemical hypercortisolism (21, 22). In this study, routine blood count-derived immune markers, particularly eosinophil depletion and increased NLR, were consistently associated with 24h-UFC, midnight serum cortisol, and 8 a.m. serum cortisol. Individual immune markers were also associated with selected electrolyte, metabolic, and skeletal complications, while higher CRIS was associated with selected complication-domain burden.

Assessment of disease severity in Cushing’s syndrome remains challenging because biochemical and clinical severity do not always coincide (23–25), and patients may show substantial heterogeneity in comorbidity burden despite similar biochemical evidence of cortisol excess (23, 26, 27). We therefore considered biochemical cortisol measures and selected complication-domain burden as related but distinct features of the clinical phenotype. Although the Cushing’s Syndrome Severity Index (CSI) is a validated multidomain measure of overall clinical severity (24, 28), several of its components require graded clinical assessment that could not be reliably reconstructed retrospectively. We therefore used an exploratory four-domain score comprising electrolyte involvement, hypertension, diabetes, and fracture, selected because these domains could be systematically ascertained from the available records. This score was intended to summarise the number of affected domains and not to replace the CSI or serve as a validated measure of overall disease severity.

Altered leukocyte profiles are well recognised in Cushing’s syndrome. Glucocorticoid excess promotes neutrophilia, lymphocyte reduction, and eosinophil depletion, changes that have been associated with cortisol secretion, disease severity, infection risk, and remission status (29–32). In our cohort, total WBC did not differ across aetiological groups, whereas lymphocyte and eosinophil counts, NLR, SIRI, and PNI showed clearer differences, particularly in EAS. These findings suggest that differential leukocyte counts and derived indices may better capture cortisol-related immune alterations than total WBC.

AEC and NLR showed the most consistent associations with the cortisol measures examined. Inflammation-based indices have previously been studied in the diagnosis and assessment of remission of Cushing’s syndrome (31, 33). Other studies have described subtype-specific leukocyte patterns (34), linked inflammation-based indices to the degree of cortisol excess (35), and examined inflammatory indices and PNI in relation to perioperative infection (36). Our findings on cortisol-related immune changes are consistent with these observations. We further examined associations with prevalent electrolyte abnormalities, diabetes, and fracture after adjustment for 24h-UFC, extending the evaluation of these markers across several complication domains. The associations of AEC with 24h-UFC and 8AM serum cortisol were also consistent across alternative log offsets and categorical analyses.

In the primary 24h-UFC–adjusted analyses, associations with immune markers were most widespread for electrolyte abnormalities. Eosinophil depletion was associated with hypokalaemia and hypophosphataemia but showed no clear association with diabetes or fracture despite its consistent relationship with cortisol. For diabetes, only SII remained significant after FDR correction. Previous studies have related eosinophil counts to glucose and HbA1c in adrenal Cushing’s syndrome and neutrophil percentage to diabetes (37, 38). Differences in aetiology, marker definitions, glycaemic outcomes, and cortisol adjustment limit direct comparison with these studies. Lower PNI was associated with fracture. As PNI combines serum albumin and lymphocyte counts, this association cannot be attributed specifically to nutritional impairment. Left ventricular remodelling has been documented in active Cushing’s syndrome (39), but none of the immune-marker associations with increased RWT remained significant after FDR correction in the primary 24h-UFC–adjusted analysis.

These findings suggest that markers associated with cortisol may not capture the full range of clinical complications. 24h-UFC shows substantial within-patient variability (25), and measurements at the index evaluation may not fully reflect the duration and magnitude of prior cortisol exposure. Associations persisting after 24h-UFC adjustment should therefore not be interpreted as evidence of cortisol-independent effects. Likewise, the exploratory mediation analyses do not establish that immune alterations cause electrolyte complications; the observed associations remain compatible with cortisol excess affecting immune markers and complications through separate pathways.

Previous studies have evaluated individual blood count-derived markers in Cushing’s syndrome (37, 38, 40). CRIS was associated with selected complication-domain burden. However, in patients with ACA or CD, it showed similar discrimination to log-NLR for multidomain complication involvement, and adding CRIS to a model containing log-NLR did not significantly improve model fit. Correlations among its components, together with the shared lymphocyte component of NLR and PNI, suggest overlapping information. CRIS therefore remains an exploratory summary of immune alterations, with no demonstrated incremental value over log–NLR in this subgroup. Its clinical utility requires external and prospective evaluation.

From a clinical perspective, routinely available blood count-derived immune markers may provide complementary information when characterising cortisol-related immune changes and complication burden in patients with established Cushing’s syndrome. These markers are inexpensive and widely available, but they are nonspecific and their potential clinical utility remains hypothesis-generating. They should not replace established endocrine, imaging, or clinical assessments.

A major strength of this study is the assessment of both biochemical and clinical correlates of routine immune markers in a cohort of 202 patients spanning different aetiologies of endogenous Cushing’s syndrome. Combining 24h-UFC with midnight serum cortisol provided complementary information on daily cortisol excretion and nocturnal cortisol elevation. The evaluation of individual complications, with adjustment for 24h-UFC, further clarified how the clinical associations of these markers varied across electrolyte, metabolic, and skeletal outcomes. This provides a clinical context for interpreting cortisol-related changes in routinely available blood tests. The broadly consistent findings across analyses addressing missing data, infection, and the AEC floor effect provide additional support for the observed association patterns.

Several limitations should be considered. The single-centre retrospective design limits generalisability and precludes temporal or causal inference, while the small EAS subgroup and limited event counts for some complications reduce the precision of the estimates. Data availability also constrained the analyses: 24h-UFC and echocardiographic measurements were incomplete, and post-dexamethasone cortisol and late-night salivary cortisol were not systematically available. Similarly, retrospective outcome ascertainment restricted the burden score to selected complications, with limited information on diabetes timing and no systematic screening for asymptomatic vertebral fractures. Although sensitivity analyses yielded similar results, the floor effect of AEC measurements remains a consideration. Finally, external validation is lacking, and the cohort-specific upper-quartile cortisol thresholds should not be interpreted as established clinical cut-offs.

## 5 Conclusion

In conclusion, routine blood-based immune markers were associated with complementary biochemical measures of cortisol excess and selected clinical complications in patients with endogenous Cushing’s syndrome. Eosinophil depletion and increased NLR were consistently associated with all three cortisol measures, whereas associations with clinical complications varied by marker and outcome. CRIS provided an exploratory integrative summary of cortisol-related immune alterations and selected complication-domain burden, but requires external validation before clinical application.

## Data Availability

The original contributions presented in the study are included in the article/**Supplementary Material**. Further inquiries can be directed to the corresponding authors.
