# Supplementary material for: Routine blood-based immune signatures in endogenous Cushing’s syndrome: associations with cortisol excess and selected complication burden
Source: Front Endocrinol (Lausanne). 2026 Oct 22;17:1961986. doi: 10.3389/fendo.2026.1961986 (PMC13735581; doi:10.3389/fendo.2026.1961986)
Supplement: Supplementary file 1 [file SupplementaryFile1.docx]

**Supplementary Table 1. Availability of key variables and analysis datasets**

| **Variable / analysis dataset** | **Available n** | **Missing n** | **Missing (%)** |
| --- | --- | --- | --- |
| Total study cohort | 202 | 0 | 0.0 |
| Aetiological classification | 202 | 0 | 0.0 |
| Complete baseline blood count-derived immune marker panel | 199 | 3 | 1.5 |
| Midnight serum cortisol available at index evaluation | 177 | 25 | 12.4 |
| 8AM serum cortisol available for cortisol analyses | 193 | 9 | 4.5 |
| 24h-UFC available for cortisol analyses | 173 | 29 | 14.4 |
| Common complete-case dataset for upper-quartile 8AM serum cortisol ROC models | 184 | 18 | 8.9 |
| Common complete-case dataset for upper-quartile 24h-UFC ROC models | 164 | 38 | 18.8 |
| Electrolyte-domain involvement | 185 | 17 | 8.4 |
| Hypertension | 202 | 0 | 0.0 |
| Diabetes | 202 | 0 | 0.0 |
| Fracture | 202 | 0 | 0.0 |
| Echocardiographic RWT | 171 | 31 | 15.3 |
| Selected complication-domain burden score | 185 | 17 | 8.4 |
| CRIS and selected complication-domain burden score available | 182 | 20 | 9.9 |
| Primary CRIS quartile disease-burden dataset used for Table 4 | 182 | 20 | 9.9 |

Missing percentages were calculated using the total cohort of 202 patients as the denominator. The complete baseline blood count-derived immune marker panel included the variables required to calculate the principal immune indices and exploratory CRIS. The common complete-case ROC datasets required available outcome status, age, sex, aetiology, serum potassium, systolic blood pressure, log-transformed NLR, eosinophil depletion, and low PNI; all three models for each outcome were fitted in the same patients. The selected complication-domain burden score included four domains: electrolyte involvement, hypertension, diabetes, and fracture. Electrolyte-domain involvement was defined as hypokalaemia and/or hypophosphataemia. One point was assigned for each affected domain, yielding a score ranging from 0 to 4. CRIS was calculated as [Z(log NLR) + Z(−log(AEC + 0.01)) + Z(−PNI)]/3. AEC, absolute eosinophil count; CRIS, cortisol-related immune score; NLR, neutrophil-to-lymphocyte ratio; PNI, prognostic nutritional index; ROC, receiver operating characteristic; RWT, relative wall thickness; UFC, urinary free cortisol.

**Supplementary Table 2. Characteristics of patients according to availability of 24-hour urinary free cortisol**

| **Variable** | **24h-UFC available (n = 173)** | **24h-UFC unavailable (n = 29)** | **P value** |
| --- | --- | --- | --- |
| Age, years | 47 (32, 54) | 43 (31, 52) | 0.420 |
| Female sex, n/N (%) | 151/173 (87.3) | 25/29 (86.2) | 0.772 |
| Aetiology, n/N (%) |  |  | <0.001 |
| ACA | 49/173 (28.3) | 22/29 (75.9) |  |
| CD | 116/173 (67.1) | 6/29 (20.7) |  |
| EAS | 8/173 (4.6) | 1/29 (3.4) |  |
| 8AM serum cortisol, nmol/L | 539.20 (400.60, 747.30) | 604.95 (501.69, 727.92) | 0.564 |
| Albumin, g/L | 38.70 (35.90, 41.50) | 37.65 (34.75, 40.62) | 0.316 |
| AEC, ×10⁹/L | 0.03 (0.01, 0.08) | 0.03 (0.01, 0.06) | 0.786 |
| NLR | 3.68 (2.41, 5.35) | 3.13 (2.35, 4.60) | 0.313 |
| PNI | 47.05 (42.75, 51.42) | 46.23 (40.14, 49.53) | 0.399 |
| Hypokalaemia, n/N (%) | 64/172 (37.2) | 11/29 (37.9) | 1.000 |
| Hypophosphataemia, n/N (%) | 19/145 (13.1) | 1/27 (3.7) | 0.207 |
| Hypertension, n/N (%) | 120/173 (69.4) | 25/29 (86.2) | 0.075 |
| Diabetes, n/N (%) | 63/173 (36.4) | 8/29 (27.6) | 0.407 |
| Fracture, n/N (%) | 55/173 (31.8) | 2/29 (6.9) | 0.006 |
| Increased RWT, n/N (%) | 22/146 (15.1) | 3/25 (12.0) | 1.000 |
| Documented infection, n/N (%) | 12/173 (6.9) | 2/29 (6.9) | 1.000 |
| Selected complication-domain burden score | 2 (1, 3) [n = 157] | 1 (1, 2) [n = 28] | 0.373 |
| Multidomain complication involvement, n/N (%) | 90/157 (57.3) | 13/28 (46.4) | 0.308 |

Continuous variables are presented as median (interquartile range), and categorical variables as number/number with available data (percentage). The selected complication-domain burden score comprised four domains: electrolyte involvement, hypertension, diabetes, and fracture, with one point assigned to each affected domain (range 0–4). Multidomain complication involvement was defined as involvement of at least two domains. Comparisons were performed using the Mann–Whitney U test for continuous variables and the χ² test or Fisher’s exact test for categorical variables, as appropriate. ACA, adrenocortical adenoma; CD, Cushing’s disease; EAS, ectopic ACTH syndrome; AEC, absolute eosinophil count; NLR, neutrophil-to-lymphocyte ratio; PNI, prognostic nutritional index; RWT, relative wall thickness; UFC, urinary free cortisol.

**Supplementary Table 3. Sensitivity analyses addressing the floor effect of absolute eosinophil count**

| **Analysis** | **Outcome** | **AEC parameterisation** | **n** | **Effect estimate (95% CI)** | **P value** |
| --- | --- | --- | --- | --- | --- |
| Cortisol association | 8AM serum cortisol | log(AEC + 0.01), primary | 193 | β −0.280 (−0.361 to −0.198) | <0.001 |
|  |  | log(AEC + 0.001) | 193 | β −0.287 (−0.369 to −0.205) | <0.001 |
|  |  | log(AEC + 0.05) | 193 | β −0.247 (−0.330 to −0.164) | <0.001 |
|  |  | AEC = 0.00 vs >0.00 ×10⁹/L | 193 | β 0.593 (0.370 to 0.815) | <0.001 |
| Cortisol association | 24h-UFC | log(AEC + 0.01), primary | 173 | β −0.687 (−0.808 to −0.565) | <0.001 |
|  |  | log(AEC + 0.001) | 173 | β −0.687 (−0.811 to −0.564) | <0.001 |
|  |  | log(AEC + 0.05) | 173 | β −0.649 (−0.774 to −0.524) | <0.001 |
|  |  | AEC = 0.00 vs >0.00 ×10⁹/L | 173 | β 1.409 (1.045 to 1.772) | <0.001 |
| Complication association | Hypokalaemia | −log(AEC + 0.01), primary | 162 | OR 2.27 (1.36–3.79) | 0.002 |
|  |  | −log(AEC + 0.001) | 162 | OR 2.08 (1.31–3.31) | 0.002 |
|  |  | −log(AEC + 0.05) | 162 | OR 2.21 (1.26–3.86) | 0.005 |
|  |  | AEC = 0.00 vs >0.00 ×10⁹/L | 162 | OR 3.24 (1.12–9.38) | 0.030 |
| Complication association | Hypophosphataemia | −log(AEC + 0.01), primary | 136 | OR 2.58 (1.26–5.53) | 0.010 |
|  |  | −log(AEC + 0.001) | 136 | OR 2.32 (1.27–4.28) | 0.007 |
|  |  | −log(AEC + 0.05) | 136 | OR 2.61 (1.12–6.94) | 0.026 |
|  |  | AEC = 0.00 vs >0.00 ×10⁹/L | 136 | OR 5.63 (1.33–24.15) | 0.020 |
| Complication association | Diabetes | −log(AEC + 0.01), primary | 163 | OR 1.00 (0.63–1.60) | 0.985 |
|  |  | −log(AEC + 0.001) | 163 | OR 1.09 (0.71–1.69) | 0.691 |
|  |  | −log(AEC + 0.05) | 163 | OR 0.97 (0.60–1.55) | 0.886 |
|  |  | AEC = 0.00 vs >0.00 ×10⁹/L | 163 | OR 1.62 (0.56–4.66) | 0.371 |
| Complication association | Fracture | −log(AEC + 0.01), primary | 163 | OR 1.30 (0.82–2.05) | 0.260 |
|  |  | −log(AEC + 0.001) | 163 | OR 1.19 (0.78–1.82) | 0.416 |
|  |  | −log(AEC + 0.05) | 163 | OR 1.31 (0.81–2.11) | 0.266 |
|  |  | AEC = 0.00 vs >0.00 ×10⁹/L | 163 | OR 0.97 (0.34–2.77) | 0.958 |
| Complication association | Increased RWT | −log(AEC + 0.01), primary | 138 | OR 0.76 (0.38–1.47) | 0.425 |
|  |  | −log(AEC + 0.001) | 138 | OR 0.94 (0.47–1.70) | 0.847 |
|  |  | −log(AEC + 0.05) | 138 | OR 0.73 (0.38–1.40) | 0.336 |
|  |  | AEC = 0.00 vs >0.00 ×10⁹/L | 138 | OR 1.75 (0.41–6.51) | 0.429 |

AEC was recorded as 0.00 ×10⁹/L in 35 of 202 patients (17.3%) and as 0.01 ×10⁹/L in 25 patients (12.4%); thus, 60 patients (29.7%) had AEC ≤0.01 ×10⁹/L. The offset of 0.01 was used in the primary analyses. For continuous AEC parameterisations, effect estimates are reported per 1-SD increase in the transformed variable. AEC was modelled as log(AEC + offset) in the cortisol analyses and as eosinophil depletion [−log(AEC + offset)] in the complication analyses. The categorical sensitivity analysis compared AEC recorded as 0.00 ×10⁹/L with AEC >0.00 ×10⁹/L. Cortisol models were adjusted for age, sex, and aetiology. Complication models excluded patients with ectopic ACTH syndrome and were additionally adjusted for log-transformed 24h-UFC (×ULN), consistent with the primary complication-analysis framework. Conventional logistic regression was used for hypokalaemia, diabetes, and fracture, whereas Firth penalised logistic regression was used for hypophosphataemia and increased RWT because of the limited number of events. AEC, absolute eosinophil count; UFC, urinary free cortisol; RWT, relative wall thickness; OR, odds ratio; CI, confidence interval; ULN, upper limit of normal.

**Supplementary Table 4. Calendar-period sensitivity analyses of associations between immune markers and cortisol measures**

| **Outcome** | **Marker** | **2012–2019, n** | **Adjusted β (95% CI)** | **P value** | **2020–2022, n** | **Adjusted β (95% CI)** | **P value** | **P for interaction** | **FDR q for interaction** |
| --- | --- | --- | --- | --- | --- | --- | --- | --- | --- |
| **8AM serum cortisol** | ANC | 103 | 0.164 (0.034 to 0.294) | 0.014 | 90 | 0.282 (0.185 to 0.379) | <0.001 | 0.251 | 0.515 |
|  | ALC | 103 | −0.174 (−0.309 to −0.038) | 0.013 | 90 | −0.304 (−0.394 to −0.213) | <0.001 | 0.075 | 0.434 |
|  | AEC | 103 | −0.297 (−0.424 to −0.169) | <0.001 | 90 | −0.268 (−0.367 to −0.170) | <0.001 | 0.368 | 0.515 |
|  | NLR | 103 | 0.265 (0.133 to 0.397) | <0.001 | 90 | 0.360 (0.278 to 0.442) | <0.001 | 0.636 | 0.696 |
|  | SII | 103 | 0.212 (0.083 to 0.342) | 0.002 | 90 | 0.341 (0.254 to 0.428) | <0.001 | 0.363 | 0.515 |
|  | SIRI | 103 | 0.190 (0.060 to 0.319) | 0.004 | 90 | 0.351 (0.265 to 0.438) | <0.001 | 0.107 | 0.434 |
|  | PNI | 102 | −0.186 (−0.328 to −0.044) | 0.011 | 89 | −0.266 (−0.373 to −0.160) | <0.001 | 0.331 | 0.515 |
| **24h-UFC** | ANC | 94 | 0.303 (0.113 to 0.492) | 0.002 | 79 | 0.682 (0.480 to 0.884) | <0.001 | 0.047 | 0.434 |
|  | ALC | 94 | −0.393 (−0.588 to −0.198) | <0.001 | 79 | −0.513 (−0.731 to −0.294) | <0.001 | 0.300 | 0.515 |
|  | AEC | 94 | −0.610 (−0.774 to −0.447) | <0.001 | 79 | −0.757 (−0.945 to −0.570) | <0.001 | 0.494 | 0.629 |
|  | NLR | 94 | 0.516 (0.337 to 0.696) | <0.001 | 79 | 0.767 (0.582 to 0.952) | <0.001 | 0.658 | 0.696 |
|  | SII | 94 | 0.385 (0.198 to 0.573) | <0.001 | 79 | 0.734 (0.539 to 0.928) | <0.001 | 0.272 | 0.515 |
|  | SIRI | 94 | 0.364 (0.174 to 0.554) | <0.001 | 79 | 0.723 (0.530 to 0.917) | <0.001 | 0.124 | 0.434 |
|  | PNI | 93 | −0.427 (−0.622 to −0.233) | <0.001 | 78 | −0.456 (−0.702 to −0.210) | <0.001 | 0.696 | 0.696 |

Multivariable linear regression models were adjusted for age, sex, and aetiology. Cortisol measures, NLR, SII, and SIRI were natural-log transformed; AEC was modelled as log(AEC + 0.01). Regression coefficients represent changes in log-transformed cortisol measures per 1-SD increase in each immune marker within each calendar period. Calendar periods were defined as 2012–2019 and 2020–2022, approximately balancing the cohort around the median admission year. P values for interaction were obtained from pooled models including a marker × calendar-period interaction term, with adjustment for age, sex, and aetiology. Benjamini–Hochberg false-discovery rate correction was applied across the 14 interaction tests. None of the interaction terms remained significant after false-discovery rate correction. ANC, absolute neutrophil count; ALC, absolute lymphocyte count; AEC, absolute eosinophil count; NLR, neutrophil-to-lymphocyte ratio; SII, systemic immune-inflammation index; SIRI, systemic inflammation response index; PNI, prognostic nutritional index; UFC, urinary free cortisol; CI, confidence interval.

**Supplementary Table 5. Discrimination, calibration, and internal validation of exploratory ROC models for upper-quartile cortisol values**

| **Outcome** | **Model** | **n** | **Apparent AUC (95% CI)** | **Optimism** | **Optimism-corrected AUC** | **Optimism-corrected calibration slope** | **Optimism-corrected Brier score** | **ΔAUC vs clinical model (P value)** |
| --- | --- | --- | --- | --- | --- | --- | --- | --- |
| Upper-quartile 8AM serum cortisol | Clinical model | 184 | 0.793 (0.717–0.869) | 0.032 | 0.761 | 0.776 | 0.156 | Reference |
|  | Immune model | 184 | 0.857 (0.794–0.920) | 0.009 | 0.849 | 0.940 | 0.129 | 0.064 (P = 0.098) |
|  | Clinical + immune model | 184 | 0.896 (0.839–0.952) | 0.030 | 0.865 | 0.752 | 0.120 | 0.102 (P < 0.001) |
| Upper-quartile 24h-UFC | Clinical model | 164 | 0.787 (0.706–0.868) | 0.036 | 0.751 | 0.633 | 0.160 | Reference |
|  | Immune model | 164 | 0.857 (0.793–0.920) | 0.009 | 0.848 | 0.941 | 0.137 | 0.070 (P = 0.135) |
|  | Clinical + immune model | 164 | 0.898 (0.847–0.950) | 0.033 | 0.866 | 0.682 | 0.132 | 0.112 (P = 0.001) |

All three models for each outcome were evaluated in the same complete-case sample. The clinical model included age, sex, aetiology, serum potassium, and systolic blood pressure; the immune model included log-transformed NLR, eosinophil depletion, and low PNI; and the clinical + immune model included both sets of variables. AUCs were compared using paired DeLong tests. Internal validation used 1,000 bootstrap resamples. Comparisons of the clinical + immune model with the immune model yielded ΔAUC = 0.039 (P = 0.041) for upper-quartile 8AM serum cortisol and ΔAUC = 0.042 (P = 0.033) for upper-quartile 24h-UFC. AEC, absolute eosinophil count; AUC, area under the receiver operating characteristic curve; CI, confidence interval; NLR, neutrophil-to-lymphocyte ratio; PNI, prognostic nutritional index; UFC, urinary free cortisol.

**Supplementary Table 6. Sensitivity analyses of ROC performance after excluding EAS and in CD-only patients**

| **Analysis** | **Outcome** | **n** | **Clinical model AUC** | **Immune model AUC** | **Clinical + immune model AUC** |
| --- | --- | --- | --- | --- | --- |
| Excluding EAS | Upper-quartile 8AM serum cortisol | 175 | 0.765 | 0.845 | 0.886 |
| Excluding EAS | Upper-quartile 24h-UFC | 156 | 0.756 | 0.843 | 0.887 |
| CD only | Upper-quartile 8AM serum cortisol | 111 | 0.755 | 0.900 | 0.929 |
| CD only | Upper-quartile 24h-UFC | 109 | 0.719 | 0.870 | 0.894 |

Sensitivity analyses were performed after excluding patients with ectopic ACTH syndrome and within the Cushing’s disease-only subgroup. The upper-quartile thresholds (≥747.30 nmol/L for 8AM serum cortisol and ≥4.11 ×ULN for 24h-UFC) were defined in the full cohort and retained unchanged in all sensitivity analyses. The clinical model included age, sex, serum potassium, systolic blood pressure, and aetiology where applicable; aetiology was omitted from the CD-only analysis. The immune model included log-transformed NLR, eosinophil depletion, and low PNI, and the clinical + immune model included both sets of variables. All three models for each outcome were evaluated in the same complete-case sample. AUC, area under the receiver operating characteristic curve; CD, Cushing’s disease; EAS, ectopic ACTH syndrome; NLR, neutrophil-to-lymphocyte ratio; PNI, prognostic nutritional index; ROC, receiver operating characteristic; UFC, urinary free cortisol.

**Supplementary Table 7. Associations of individual immune markers with selected complications in primary and sensitivity analyses**

**Panel A. Primary analysis adjusted for 24h-UFC**

| **Outcome** | **Immune marker** | **n** | **Events** | **OR (95% CI)** | **P value** | **FDR q value** |
| --- | --- | --- | --- | --- | --- | --- |
| Hypokalaemia | Log-NLR | 162 | 56 | 1.80 (1.15–2.83) | 0.011 | 0.028 |
|  | AEC depletion | 162 | 56 | 2.27 (1.36–3.79) | 0.002 | 0.016 |
|  | Log-SII | 162 | 56 | 1.79 (1.14–2.81) | 0.011 | 0.028 |
|  | Log-SIRI | 162 | 56 | 1.40 (0.93–2.12) | 0.106 | 0.133 |
|  | Low PNI | 162 | 56 | 1.70 (1.12–2.60) | 0.013 | 0.031 |
| Hypophosphataemia | Log-NLR | 136 | 17 | 2.86 (1.39–6.33) | 0.004 | 0.023 |
|  | AEC depletion | 136 | 17 | 2.58 (1.26–5.53) | 0.010 | 0.028 |
|  | Log-SII | 136 | 17 | 1.87 (0.95–3.84) | 0.070 | 0.092 |
|  | Log-SIRI | 136 | 17 | 2.15 (1.08–4.69) | 0.029 | 0.055 |
|  | Low PNI | 136 | 17 | 2.29 (1.23–4.57) | 0.009 | 0.028 |
| Diabetes | Log-NLR | 163 | 58 | 1.54 (0.99–2.40) | 0.055 | 0.087 |
|  | AEC depletion | 163 | 58 | 1.00 (0.63–1.60) | 0.985 | 0.985 |
|  | Log-SII | 163 | 58 | 1.94 (1.23–3.07) | 0.005 | 0.023 |
|  | Log-SIRI | 163 | 58 | 1.52 (1.00–2.32) | 0.052 | 0.086 |
|  | Low PNI | 163 | 58 | 1.18 (0.80–1.74) | 0.403 | 0.438 |
| Fracture | Log-NLR | 163 | 53 | 2.03 (1.30–3.18) | 0.002 | 0.016 |
|  | AEC depletion | 163 | 53 | 1.30 (0.82–2.05) | 0.260 | 0.296 |
|  | Log-SII | 163 | 53 | 1.75 (1.14–2.69) | 0.011 | 0.028 |
|  | Log-SIRI | 163 | 53 | 1.58 (1.05–2.36) | 0.027 | 0.055 |
|  | Low PNI | 163 | 53 | 2.52 (1.60–3.96) | <0.001 | 0.001 |
| Increased RWT | Log-NLR | 138 | 22 | 1.68 (0.96–2.96) | 0.067 | 0.092 |
|  | AEC depletion | 138 | 22 | 0.76 (0.38–1.47) | 0.425 | 0.443 |
|  | Log-SII | 138 | 22 | 1.70 (0.97–3.06) | 0.063 | 0.092 |
|  | Log-SIRI | 138 | 22 | 1.46 (0.87–2.51) | 0.152 | 0.181 |
|  | Low PNI | 138 | 22 | 1.69 (1.01–2.90) | 0.046 | 0.082 |

**Panel B. Models without 24h-UFC adjustment in the same complete-case samples**

| **Outcome** | **Immune marker** | **n** | **Events** | **OR (95% CI)** | **P value** | **FDR q value** |
| --- | --- | --- | --- | --- | --- | --- |
| Hypokalaemia | Log-NLR | 162 | 56 | 2.14 (1.47–3.11) | <0.001 | <0.001 |
|  | AEC depletion | 162 | 56 | 2.51 (1.68–3.76) | <0.001 | <0.001 |
|  | Log-SII | 162 | 56 | 2.16 (1.46–3.20) | <0.001 | <0.001 |
|  | Log-SIRI | 162 | 56 | 1.77 (1.24–2.53) | 0.002 | 0.004 |
|  | Low PNI | 162 | 56 | 2.06 (1.39–3.06) | <0.001 | 0.001 |
| Hypophosphataemia | Log-NLR | 136 | 17 | 2.76 (1.56–5.40) | <0.001 | 0.001 |
|  | AEC depletion | 136 | 17 | 2.58 (1.47–4.90) | <0.001 | 0.002 |
|  | Log-SII | 136 | 17 | 2.17 (1.26–4.02) | 0.005 | 0.010 |
|  | Log-SIRI | 136 | 17 | 2.44 (1.37–4.83) | 0.002 | 0.004 |
|  | Low PNI | 136 | 17 | 2.61 (1.47–5.00) | <0.001 | 0.003 |
| Diabetes | Log-NLR | 163 | 58 | 1.53 (1.08–2.17) | 0.016 | 0.026 |
|  | AEC depletion | 163 | 58 | 1.20 (0.85–1.69) | 0.303 | 0.316 |
|  | Log-SII | 163 | 58 | 1.83 (1.25–2.68) | 0.002 | 0.004 |
|  | Log-SIRI | 163 | 58 | 1.55 (1.09–2.22) | 0.016 | 0.026 |
|  | Low PNI | 163 | 58 | 1.29 (0.90–1.84) | 0.169 | 0.192 |
| Fracture | Log-NLR | 163 | 53 | 1.63 (1.15–2.30) | 0.006 | 0.011 |
|  | AEC depletion | 163 | 53 | 1.23 (0.88–1.73) | 0.228 | 0.247 |
|  | Log-SII | 163 | 53 | 1.54 (1.08–2.18) | 0.016 | 0.026 |
|  | Log-SIRI | 163 | 53 | 1.45 (1.03–2.04) | 0.032 | 0.047 |
|  | Low PNI | 163 | 53 | 2.24 (1.50–3.35) | <0.001 | <0.001 |
| Increased RWT | Log-NLR | 138 | 22 | 1.48 (0.94–2.39) | 0.093 | 0.116 |
|  | AEC depletion | 138 | 22 | 0.91 (0.55–1.48) | 0.694 | 0.694 |
|  | Log-SII | 138 | 22 | 1.52 (0.95–2.50) | 0.082 | 0.108 |
|  | Log-SIRI | 138 | 22 | 1.39 (0.88–2.25) | 0.156 | 0.186 |
|  | Low PNI | 138 | 22 | 1.62 (1.00–2.69) | 0.048 | 0.067 |

**Panel C. Primary 24h-UFC-adjusted analysis after excluding patients with documented infection**

| **Outcome** | **Immune marker** | **n** | **Events** | **OR (95% CI)** | **P value** | **FDR q value** |
| --- | --- | --- | --- | --- | --- | --- |
| Hypokalaemia | Log-NLR | 153 | 51 | 1.78 (1.11–2.85) | 0.017 | 0.041 |
|  | AEC depletion | 153 | 51 | 2.55 (1.48–4.41) | <0.001 | 0.007 |
|  | Log-SII | 153 | 51 | 1.72 (1.07–2.76) | 0.024 | 0.047 |
|  | Log-SIRI | 153 | 51 | 1.26 (0.82–1.94) | 0.296 | 0.336 |
|  | Low PNI | 153 | 51 | 1.97 (1.26–3.10) | 0.003 | 0.013 |
| Hypophosphataemia | Log-NLR | 131 | 15 | 2.48 (1.17–5.62) | 0.018 | 0.041 |
|  | AEC depletion | 131 | 15 | 3.02 (1.42–6.87) | 0.004 | 0.013 |
|  | Log-SII | 131 | 15 | 1.57 (0.76–3.30) | 0.221 | 0.276 |
|  | Log-SIRI | 131 | 15 | 1.71 (0.83–3.77) | 0.146 | 0.193 |
|  | Low PNI | 131 | 15 | 2.72 (1.39–5.83) | 0.003 | 0.013 |
| Diabetes | Log-NLR | 154 | 55 | 1.60 (1.01–2.55) | 0.046 | 0.072 |
|  | AEC depletion | 154 | 55 | 1.06 (0.66–1.71) | 0.815 | 0.815 |
|  | Log-SII | 154 | 55 | 2.08 (1.27–3.40) | 0.004 | 0.013 |
|  | Log-SIRI | 154 | 55 | 1.62 (1.03–2.54) | 0.035 | 0.058 |
|  | Low PNI | 154 | 55 | 1.22 (0.81–1.82) | 0.339 | 0.369 |
| Fracture | Log-NLR | 154 | 52 | 2.38 (1.47–3.85) | <0.001 | 0.006 |
|  | AEC depletion | 154 | 52 | 1.31 (0.82–2.10) | 0.256 | 0.305 |
|  | Log-SII | 154 | 52 | 2.01 (1.27–3.19) | 0.003 | 0.013 |
|  | Log-SIRI | 154 | 52 | 1.86 (1.20–2.89) | 0.006 | 0.016 |
|  | Low PNI | 154 | 52 | 2.64 (1.65–4.21) | <0.001 | 0.001 |
| Increased RWT | Log-NLR | 132 | 22 | 1.91 (1.07–3.50) | 0.028 | 0.050 |
|  | AEC depletion | 132 | 22 | 0.77 (0.38–1.50) | 0.444 | 0.462 |
|  | Log-SII | 132 | 22 | 1.96 (1.09–3.65) | 0.025 | 0.047 |
|  | Log-SIRI | 132 | 22 | 1.65 (0.96–2.93) | 0.070 | 0.098 |
|  | Low PNI | 132 | 22 | 1.63 (0.98–2.80) | 0.061 | 0.090 |

Odds ratios are reported per 1-SD increase in each risk-oriented immune marker. All analyses excluded patients with ectopic ACTH syndrome. Panels A and B were fitted in identical complete-case samples to permit direct comparison before and after adjustment for 24h-UFC. Panel A, designated as the primary analysis, was adjusted for age, sex, aetiology, and log-transformed 24h-UFC (×ULN), whereas Panel B omitted 24h-UFC from an otherwise identical model. Panel C repeated the primary 24h-UFC-adjusted analysis after excluding patients with documented infection. Conventional logistic regression was used for hypokalaemia, diabetes, and fracture, whereas Firth penalised logistic regression was used for hypophosphataemia and increased RWT because of the limited number of events. Events per predictor variable in the primary models were 11.2 for hypokalaemia, 3.4 for hypophosphataemia, 11.6 for diabetes, 10.6 for fracture, and 4.4 for increased RWT. Benjamini–Hochberg false-discovery rate correction was applied across the 25 prespecified marker–outcome comparisons within each panel. AEC, absolute eosinophil count; FDR, false-discovery rate; NLR, neutrophil-to-lymphocyte ratio; PNI, prognostic nutritional index; RWT, relative wall thickness; SII, systemic immune-inflammation index; SIRI, systemic inflammation response index; UFC, urinary free cortisol; ULN, upper limit of normal; OR, odds ratio; CI, confidence interval.

**Supplementary Table 8. Multiple-imputation sensitivity analyses of 24h-UFC-adjusted associations between immune markers and selected complications**

| **Outcome** | **Immune marker** | **Complete-case n** | **Complete-case OR (95% CI)** | **MI n** | **MI pooled OR (95% CI)** | **MI P value** | **MI FDR q value** |
| --- | --- | --- | --- | --- | --- | --- | --- |
| Hypokalaemia | Log-NLR | 162 | 1.80 (1.15–2.83) | 189 | 1.67 (1.10–2.54) | 0.017 | 0.039 |
|  | AEC depletion | 162 | 2.27 (1.36–3.79) | 189 | 1.97 (1.24–3.12) | 0.004 | 0.020 |
|  | Log-SII | 162 | 1.79 (1.14–2.81) | 189 | 1.63 (1.08–2.48) | 0.021 | 0.040 |
|  | Log-SIRI | 162 | 1.40 (0.93–2.12) | 189 | 1.38 (0.93–2.04) | 0.109 | 0.144 |
|  | Low PNI | 162 | 1.70 (1.12–2.60) | 189 | 1.70 (1.16–2.50) | 0.006 | 0.027 |
| Hypophosphataemia | Log-NLR | 136 | 2.86 (1.39–6.33) | 162 | 3.00 (1.45–6.22) | 0.003 | 0.019 |
|  | AEC depletion | 136 | 2.58 (1.26–5.53) | 162 | 2.26 (1.16–4.42) | 0.017 | 0.039 |
|  | Log-SII | 136 | 1.87 (0.95–3.84) | 162 | 1.90 (0.99–3.66) | 0.054 | 0.091 |
|  | Log-SIRI | 136 | 2.15 (1.08–4.69) | 162 | 1.99 (1.01–3.95) | 0.048 | 0.086 |
|  | Low PNI | 136 | 2.29 (1.23–4.57) | 162 | 2.08 (1.15–3.78) | 0.016 | 0.039 |
| Diabetes | Log-NLR | 163 | 1.54 (0.99–2.40) | 190 | 1.48 (0.97–2.24) | 0.069 | 0.096 |
|  | AEC depletion | 163 | 1.00 (0.63–1.60) | 190 | 0.94 (0.61–1.43) | 0.764 | 0.764 |
|  | Log-SII | 163 | 1.94 (1.23–3.07) | 190 | 1.94 (1.25–3.02) | 0.003 | 0.019 |
|  | Log-SIRI | 163 | 1.52 (1.00–2.32) | 190 | 1.68 (1.11–2.56) | 0.014 | 0.039 |
|  | Low PNI | 163 | 1.18 (0.80–1.74) | 190 | 1.15 (0.81–1.64) | 0.436 | 0.495 |
| Fracture | Log-NLR | 163 | 2.03 (1.30–3.18) | 190 | 2.06 (1.34–3.18) | 0.001 | 0.013 |
|  | AEC depletion | 163 | 1.30 (0.82–2.05) | 190 | 1.27 (0.83–1.95) | 0.264 | 0.314 |
|  | Log-SII | 163 | 1.75 (1.14–2.69) | 190 | 1.73 (1.14–2.61) | 0.010 | 0.035 |
|  | Log-SIRI | 163 | 1.58 (1.05–2.36) | 190 | 1.61 (1.08–2.39) | 0.020 | 0.040 |
|  | Low PNI | 163 | 2.52 (1.60–3.96) | 190 | 2.00 (1.34–2.98) | <0.001 | 0.013 |
| Increased RWT | Log-NLR | 138 | 1.68 (0.96–2.96) | 162 | 1.67 (0.98–2.85) | 0.059 | 0.092 |
|  | AEC depletion | 138 | 0.76 (0.38–1.47) | 162 | 0.84 (0.47–1.49) | 0.553 | 0.576 |
|  | Log-SII | 138 | 1.70 (0.97–3.06) | 162 | 1.66 (0.97–2.84) | 0.065 | 0.096 |
|  | Log-SIRI | 138 | 1.46 (0.87–2.51) | 162 | 1.49 (0.89–2.50) | 0.127 | 0.159 |
|  | Low PNI | 138 | 1.69 (1.01–2.90) | 162 | 1.18 (0.74–1.88) | 0.486 | 0.528 |

Missing 24h-UFC values were addressed using multiple imputation by chained equations with 50 imputed datasets. The imputation model included age, sex, aetiology, log-transformed 8AM serum cortisol, the five risk-oriented immune markers, the selected complication outcomes, and documented infection. Predictive mean matching was used for continuous variables and logistic regression for binary variables. Imputed outcome values were used only within the imputation procedure; regression analyses remained restricted to patients with observed outcome data.

All analyses excluded patients with ectopic ACTH syndrome and were adjusted for age, sex, aetiology, and log-transformed 24h-UFC. Odds ratios are reported per 1-SD increase in each risk-oriented immune marker. Conventional logistic regression was used for hypokalaemia, diabetes, and fracture, whereas Firth penalised logistic regression was used for hypophosphataemia and increased RWT because of the limited number of events. Estimates from the 50 imputed datasets were pooled using Rubin’s rules. Benjamini–Hochberg false-discovery rate correction was applied across the 25 immune marker–outcome comparisons. AEC, absolute eosinophil count; EAS, ectopic ACTH syndrome; MI, multiple imputation; NLR, neutrophil-to-lymphocyte ratio; PNI, prognostic nutritional index; RWT, relative wall thickness; SII, systemic immune-inflammation index; SIRI, systemic inflammation response index; UFC, urinary free cortisol; OR, odds ratio; CI, confidence interval; FDR, false-discovery rate.

**Supplementary Table 9. Exploratory mediation analyses of 24h-UFC, immune markers, and electrolyte complications**

| **Outcome** | **Potential mediator** | **n** | **Events** | **Indirect effect, percentage points (95% CI)** | **Direct effect, percentage points (95% CI)** | **Total effect, percentage points (95% CI)** |
| --- | --- | --- | --- | --- | --- | --- |
| Hypokalaemia | Log-NLR | 162 | 56 | 8.6 (2.1 to 16.7) | 6.7 (−3.8 to 19.2) | 15.3 (6.0 to 27.1) |
|  | AEC depletion | 162 | 56 | 12.2 (5.2 to 21.7) | 3.2 (−8.6 to 16.7) | 15.4 (6.3 to 27.0) |
|  | Log-SII | 162 | 56 | 7.5 (1.4 to 13.9) | 7.9 (−2.7 to 19.9) | 15.5 (5.8 to 27.6) |
|  | Log-SIRI | 162 | 56 | 4.3 (−0.8 to 10.2) | 11.0 (0.3 to 23.8) | 15.3 (5.9 to 26.7) |
|  | Low PNI | 162 | 56 | 4.7 (0.8 to 9.8) | 10.3 (0.2 to 22.7) | 15.0 (6.0 to 27.3) |
| Hypophosphataemia | Log-NLR | 136 | 17 | 7.7 (2.2 to 15.4) | −0.5 (−3.7 to 8.4) | 7.2 (1.8 to 20.2) |
|  | AEC depletion | 136 | 17 | 7.7 (0.8 to 15.2) | −0.0 (−4.0 to 9.6) | 7.7 (2.7 to 19.7) |
|  | Log-SII | 136 | 17 | 4.6 (−0.6 to 10.8) | 2.4 (−1.6 to 12.3) | 7.0 (2.1 to 18.2) |
|  | Log-SIRI | 136 | 17 | 5.5 (−0.3 to 11.5) | 1.7 (−2.2 to 13.2) | 7.2 (2.0 to 19.7) |
|  | Low PNI | 136 | 17 | 4.0 (0.7 to 7.5) | 2.5 (−1.4 to 13.1) | 6.5 (2.1 to 18.1) |

Analyses used the same common complete-case datasets as the corresponding 24h-UFC-adjusted complication models and excluded patients with ectopic ACTH syndrome. Models were adjusted for age, sex, and aetiology. Effects are expressed as average absolute changes in predicted outcome probability, in percentage points, associated with a 1-SD increase in log-transformed 24h-UFC. Indirect, direct, and total effects were estimated using model-based standardisation, with 95% confidence intervals obtained from 1,000 nonparametric bootstrap resamples. These analyses were exploratory and should not be interpreted as establishing causal mediation. AEC, absolute eosinophil count; NLR, neutrophil-to-lymphocyte ratio; PNI, prognostic nutritional index; SII, systemic immune-inflammation index; SIRI, systemic inflammation response index; UFC, urinary free cortisol; CI, confidence interval.

**Supplementary Table 10. Sensitivity and aetiology-specific analyses of associations between CRIS and disease-burden outcomes**

| **Analysis** | **Outcome** | **n** | **Effect estimate** | **95% CI** | **P value** |
| --- | --- | --- | --- | --- | --- |
| Additional adjustment for 24h-UFC | Selected complication-domain burden score | 155 | IRR 1.29 | 1.09–1.51 | 0.003 |
|  | Multidomain complication involvement | 155 | OR 2.07 | 1.23–3.71 | 0.006 |
| Excluding EAS | Selected complication-domain burden score | 173 | IRR 1.19 | 1.07–1.33 | 0.001 |
|  | Multidomain complication involvement | 173 | OR 1.82 | 1.29–2.63 | <0.001 |
| Excluding documented infection | Selected complication-domain burden score | 170 | IRR 1.22 | 1.09–1.37 | <0.001 |
|  | Multidomain complication involvement | 170 | OR 1.99 | 1.38–2.98 | <0.001 |
| CD only | Selected complication-domain burden score | 113 | IRR 1.23 | 1.08–1.40 | 0.002 |
|  | Multidomain complication involvement | 113 | OR 1.81 | 1.20–2.85 | 0.004 |
| ACA only | Selected complication-domain burden score | 60 | IRR 1.07 | 0.89–1.29 | 0.471 |
|  | Multidomain complication involvement | 60 | OR 1.57 | 0.89–2.92 | 0.121 |

Effect estimates are reported per 1-SD increase in CRIS. For the 24h-UFC-adjusted analysis, CRIS standardisation from the primary analysis was retained, and models were additionally adjusted for log-transformed 24h-UFC (×ULN). For the remaining analyses, CRIS was standardised within the corresponding dataset. Models were adjusted for age, sex, and aetiology, except for aetiology-specific analyses, which were adjusted for age and sex. The complication-domain burden score was analysed using Poisson regression, and multidomain complication involvement using Firth penalised logistic regression. CRIS, cortisol-related immune score; UFC, urinary free cortisol; ULN, upper limit of normal; EAS, ectopic ACTH syndrome; CD, Cushing’s disease; ACA, adrenocortical adenoma; OR, odds ratio; IRR, incidence rate ratio; CI, confidence interval.

**Supplementary Table 11. Robustness analyses of CRIS associations with complication-domain burden**

| **Analysis** | **Outcome** | **n** | **Effect estimate** | **95% CI** | **P value** |
| --- | --- | --- | --- | --- | --- |
| Robust SE Poisson model | Selected complication-domain burden score | 182 | IRR 1.20 | 1.10–1.31 | <0.001 |
| CRIS using AEC offset 0.001 | Selected complication-domain burden score | 182 | IRR 1.20 | 1.07–1.34 | 0.001 |
|  | Multidomain complication involvement | 182 | OR 1.95 | 1.34–2.95 | <0.001 |
| CRIS using AEC offset 0.05 | Selected complication-domain burden score | 182 | IRR 1.20 | 1.07–1.34 | 0.002 |
|  | Multidomain complication involvement | 182 | OR 1.83 | 1.28–2.69 | <0.001 |

Effect estimates are reported per 1-SD increase in the corresponding CRIS and were adjusted for age, sex, and aetiology. The selected complication-domain burden score comprised electrolyte involvement, hypertension, diabetes, and fracture (range 0–4), and multidomain complication involvement was defined as involvement of at least two domains. The robust standard error analysis was applied to the Poisson model of the burden score. For the alternative-offset analyses, CRIS was reconstructed using eosinophil depletion defined as −log(AEC + 0.001) or −log(AEC + 0.05), respectively, with the other CRIS components unchanged. The burden score was analysed using Poisson regression and multidomain complication involvement using Firth penalised logistic regression. CRIS, cortisol-related immune score; AEC, absolute eosinophil count; OR, odds ratio; IRR, incidence rate ratio; CI, confidence interval; SE, standard error.

**Supplementary Table 12. Comparison of CRIS and its individual components for multidomain complication involvement**

| **Predictor** | **Outcome** | **n** | **OR per 1-SD increase** | **95% CI** | **P value** |
| --- | --- | --- | --- | --- | --- |
| CRIS | Multidomain complication involvement | 182 | 1.90 | 1.32–2.82 | <0.001 |
| Log-NLR | Multidomain complication involvement | 182 | 1.86 | 1.29–2.75 | <0.001 |
| AEC depletion | Multidomain complication involvement | 182 | 1.43 | 1.03–2.01 | 0.031 |
| Low PNI | Multidomain complication involvement | 182 | 1.72 | 1.20–2.54 | 0.003 |

Effect estimates were calculated per 1-SD increase in each predictor using Firth penalised logistic regression and were adjusted for age, sex, and aetiology. Multidomain complication involvement was defined as involvement of at least two of four domains: electrolyte involvement, hypertension, diabetes, and fracture. CRIS was calculated as the equally weighted mean of standardised log-NLR, eosinophil depletion, and low PNI. Log-NLR was defined as the natural logarithm of NLR, eosinophil depletion as −log(AEC + 0.01), and low PNI as −PNI. All predictors were standardised within the same analysis dataset. CRIS, cortisol-related immune score; NLR, neutrophil-to-lymphocyte ratio; AEC, absolute eosinophil count; PNI, prognostic nutritional index; OR, odds ratio; CI, confidence interval.

**Supplementary Table 13. Formal comparison of CRIS with log-NLR and assessment of inter-component collinearity**

**Panel A.** **Comparison of CRIS and log-NLR for multidomain complication involvement in patients with ACA or CD**

| **Analysis** | **CRIS** | **Log-NLR** | **Comparison** |
| --- | --- | --- | --- |
| n / events | 173 / 93 | 173 / 93 | — |
| AUC (95% CI) | 0.737 (0.663–0.811) | 0.738 (0.664–0.813) | ΔAUC = −0.001; P = 0.943¹ |
| AIC | 219.46 | 220.05 | — |
| 10-fold cross-validated AUC | 0.711 | 0.706 | — |
| Incremental model fit² | — | — | χ² = 1.12; P = 0.290 |

**Panel B. Correlations and variance inflation factors among CRIS components**

| **Component** | **Log-NLR** | **AEC depletion** | **Low PNI** | **VIF** |
| --- | --- | --- | --- | --- |
| Log-NLR | 1.000 | 0.619 | 0.604 | 2.18 |
| AEC depletion | 0.619 | 1.000 | 0.381 | 1.62 |
| Low PNI | 0.604 | 0.381 | 1.000 | 1.57 |

¹ AUCs were compared using a paired DeLong test.
² Likelihood-ratio test comparing the age-, sex-, aetiology-, and log-NLR-adjusted model with the same model additionally containing CRIS.
Panel A was restricted to patients with ACA or CD because all patients with EAS met the definition of multidomain complication involvement, resulting in complete separation in conventional logistic regression. All models were adjusted for age, sex, and aetiology and evaluated in the same complete-case sample. Internal discrimination was assessed using stratified 10-fold cross-validation. Panel B was based on the full primary CRIS analysis dataset (n = 182) and shows pairwise correlations among the transformed CRIS components and variance inflation factors from a model containing all three components. CRIS was calculated as [Z(log NLR) + Z(−log(AEC + 0.01)) + Z(−PNI)]/3. ACA, adrenocortical adenoma; CD, Cushing’s disease; EAS, ectopic ACTH syndrome; AEC, absolute eosinophil count; AIC, Akaike information criterion; AUC, area under the receiver operating characteristic curve; CRIS, cortisol-related immune score; NLR, neutrophil-to-lymphocyte ratio; PNI, prognostic nutritional index; VIF, variance inflation factor.

**Supplementary Figure 1. Exploratory receiver operating characteristic analyses of clinical and immune models for upper-quartile cortisol values.**
(A) ROC curves for upper-quartile 8AM serum cortisol (n = 184). (B) ROC curves for upper-quartile 24h-UFC (n = 164). The clinical model included age, sex, aetiology, serum potassium, and systolic blood pressure. The immune model included log-NLR, eosinophil depletion, and low PNI, and the clinical + immune model included both sets of variables. All three models for each outcome were evaluated in the same complete-case sample. AUCs were compared using paired DeLong tests. Discrimination, calibration, and bootstrap internal-validation metrics are presented in Supplementary Table 5; sensitivity analyses after excluding EAS and in the Cushing’s disease-only subgroup are presented in Supplementary Table 6. AUC, area under the receiver operating characteristic curve; EAS, ectopic ACTH syndrome; NLR, neutrophil-to-lymphocyte ratio; PNI, prognostic nutritional index; ROC, receiver operating characteristic; UFC, urinary free cortisol.
